# Nicotinamide N‐Methyltransferase Epigenetically Activates Fibronectin 1 Through H3K9me3 Remodeling in Clear Cell Renal Cell Carcinoma

**DOI:** 10.1002/mco2.70831

**Published:** 2026-07-05

**Authors:** Lingling Wang, Yueyang Wang, Qizheng Han, Xiao Zhou, Chenxia Wu, Honghe Zhang, Zhiyong Liang, Maode Lai

**Affiliations:** ^1^ Department of Pathology Peking Union Medical College Hospital Chinese Academy of Medical Sciences and Peking Union Medical College Beijing China; ^2^ Research Unit of Intelligence Classification of Tumor Pathology and Precision Therapy Chinese Academy of Medical Sciences (2019RU042) and Zhejiang University School of Medicine Hangzhou China; ^3^ Department of Pathology Zhejiang University School of Medicine Hangzhou China

**Keywords:** clear cell renal cell carcinoma, fibronectin 1, metabolism, methylation, nicotinamide N‐methyltransferase, therapeutic target

## Abstract

Clear cell renal cell carcinoma (ccRCC) is among the most prevalent malignancies of the urinary system. Patients with advanced ccRCC have poor clinical outcomes. This study aimed to investigate the role of nicotinamide N‐methyltransferase (NNMT) in ccRCC. Multiomics analyses revealed aberrant expression of two enzymes in the nicotinamide metabolic pathway, both of which are associated with poor prognosis, with NNMT exhibiting the most pronounced alteration. NNMT modulation per se induces a malignant phenotype, irrespective of substrate or product supplementation. Mechanistically, using western blotting and chromatin immunoprecipitation assays, we demonstrated that NNMT decreases S‐adenosylmethionine, thereby reducing histone H3 lysine 9 trimethylation (H3K9me3) at the fibronectin 1 (FN1) promoter and activating FN1 transcription. Exogenous FN1 rescued the migration and invasion in NNMT knockout cells. In clinical specimens, NNMT expression levels were markedly elevated in tumor tissues and correlated with tumor stage. NNMT inhibitor suppressed FN1 expression and tumor progression both in vitro and in vivo. Collectively, our findings establish NNMT as a pivotal epigenetic regulator that drives ccRCC progression through extracellular matrix remodeling, providing novel early‐stage diagnostic and therapeutic strategies for ccRCC.

## Graphical abstract

Upregulation of NNMT in ccRCC is associated with tumor stage and poor prognosis. Mechanistically, NNMT reduces SAM levels, thereby decreasing H3K9me3 modification and promoting FN1 transcription. NNMT inhibitor effectively suppresses tumor progression, providing compelling evidence for its potential as a novel target for therapeutic intervention in ccRCC.

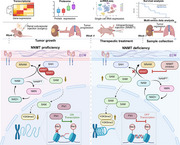

## 1 Introduction

Nicotinamide N‐methyltransferase (NNMT) is a cytoplasmic enzyme that transfers methyl from S‐adenosylmethionine (SAM) into the substrate nicotinamide (NAM) [1]. NAM is also a critical precursor of nicotinamide adenine dinucleotide (NAD), which is an essential component that couples cellular redox balance with the metabolic energy [2]. In addition to serving as a methyl donor for the NAM methylation, recent studies have identified that SAM provides methyl groups for DNA, RNA, and histone methylation [3, 4, 5]. NNMT is a novel modulator of methylation, thereby linking metabolic pathways to epigenetic regulation. NNMT has been reported to be overexpressed in multiple malignancies, such as stomach cancer [6, 7], breast cancer [8, 9], neck and head squamous cell carcinoma [10], renal cancer [11], and hepatocellular carcinoma [12]. Nevertheless, its functional role and underlying mechanisms in clear cell renal cell carcinoma (ccRCC) remain inadequately understood.

ccRCC is the most common subtype of renal malignancy, representing approximately 70%–80% of renal cell carcinomas [13]. Early diagnosis is crucial for improving patient outcomes in patients with ccRCC. Notably, approximately 15% of patients are diagnosed with stage IV disease. For patients with metastatic ccRCC, which is characterized by high aggressiveness and metastatic potential, novel therapies such as immune checkpoint inhibitors and tyrosine kinase inhibitors have shown clinical benefits [14], but their efficacy is limited by drug resistance and frequent relapse. Therefore, ccRCC remains a major clinical challenge due to therapeutic resistance and poor prognosis in advanced‐stage disease, emphasizing the need to elucidate its molecular mechanisms and identify novel diagnostic biomarkers and therapeutic targets. ccRCC exhibits significant metabolic heterogeneity. Comparative metabolomic profiling of tumor and adjacent nontumor tissues has revealed multiple dysregulated metabolic pathways in ccRCC [15, 16, 17].

The extracellular matrix (ECM), a complex network of proteins that provides structural and biochemical support to cells, is essential for maintaining tissue homeostasis [18]. ECM remodeling is a recognized facilitator of tumor invasion and metastasis and represents a key feature of ccRCC progression, as highlighted by integrative molecular analyses of this malignancy [19, 20, 21]. NNMT was shown to enhance the secretion of multiple pro‐inflammatory cytokines, collagens, chemokines, and ECM‐associated proteins [22]. Fibronectin 1 (FN1), a major ECM glycoprotein, mediates cell adhesion, migration, and signal transduction. Elevated FN1 expression has been shown to promote malignant phenotypes in various cancers. Nevertheless, the regulatory relationship between NNMT and ECM components such as FN1 in ccRCC has not been systematically explored.

Epigenetic regulation, particularly histone modifications, is being increasingly recognized for its role in cancer biology. Histone H3 lysine 9 trimethylation (H3K9me3) is a repressive mark involved in chromatin compaction and transcriptional silencing [8, 23, 24]. Previous studies suggest that metabolic enzymes such as NNMT may influence epigenetic landscapes by modulating SAM [3, 25]. In ccRCC, recent multi‐omic studies have emphasized the close interplay between metabolic states and immune microenvironmental heterogeneity [26], whereas macrophage‐driven TGF‐β signaling has been shown to promote immune suppression and resistance to immune checkpoint blockade [27]. Emerging evidence indicates that NNMT induces epigenetic reprogramming by reducing H3K27me3 levels, thereby promoting complement secretion in cancer‐associated fibroblasts and facilitating the recruitment of immunosuppressive myeloid‐derived suppressor cells, ultimately impairing antitumor immunity [17]. Collectively, these findings suggest that NNMT‐driven metabolic alterations may influence tumor progression through multifaceted mechanisms. Whether NNMT regulates ECM gene expression via epigenetic modifications in ccRCC remains to be elucidated.

In this study, we comprehensively investigated the expression patterns and functional roles of NNMT in ccRCC. Our findings reveal a critical NNMT‐driven metabolic–epigenetic axis that promotes ccRCC cell malignancy and in vivo progression, providing new perspectives on ccRCC pathogenesis and highlighting NNMT as a potential marker for diagnosis and a target for therapy.

## 2 Results

### 2.1 NNMT Upregulation in Tumors Is Linked to Adverse Prognosis in ccRCC

**FIGURE 1:**
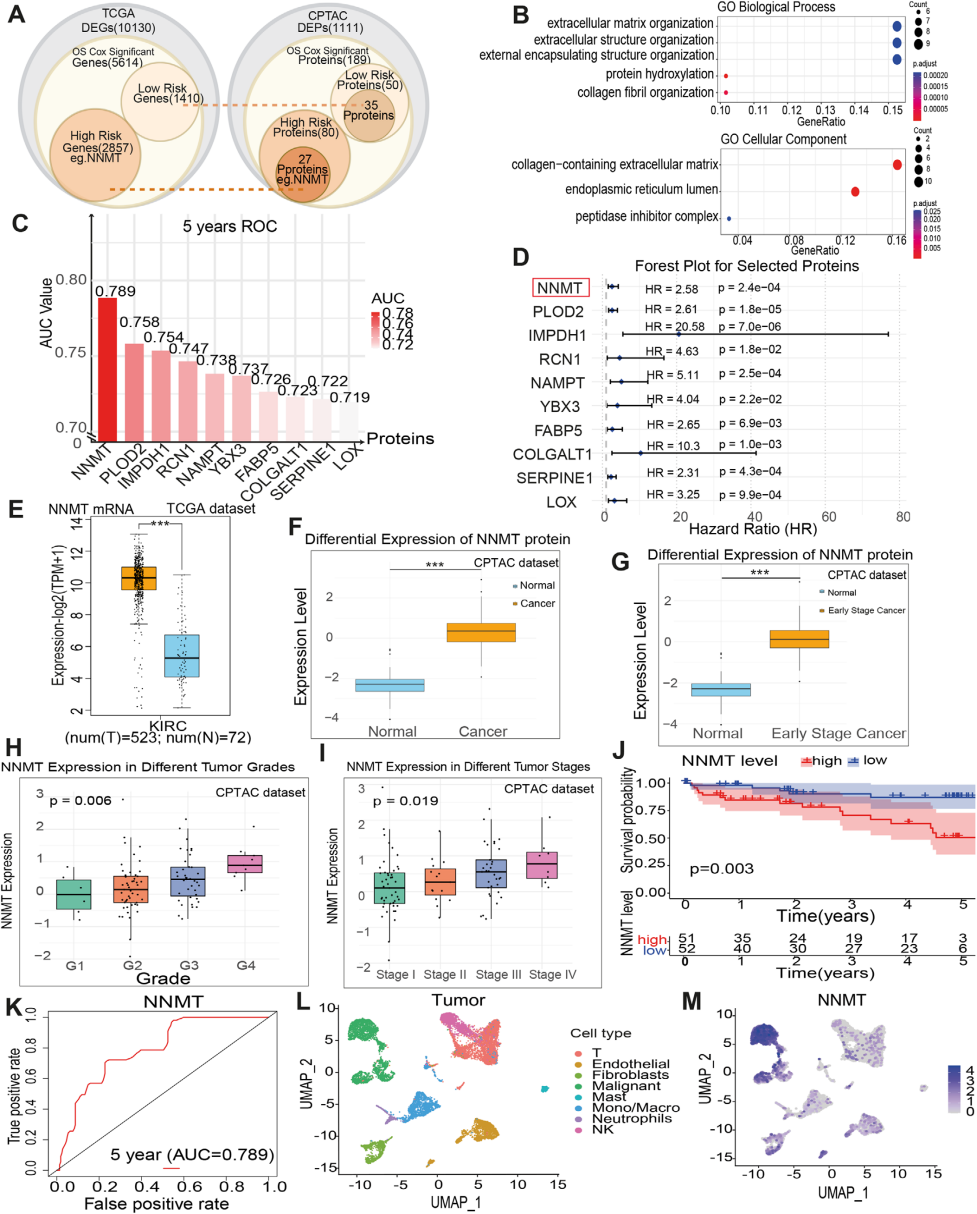
NNMT upregulation in ccRCC is associated with poor prognosis. (A) Bioinformatics analysis overview (TCGA and CPTAC datasets). (B) GO enrichment analysis of risk‐associated proteins consistently identified at both the transcriptomic and proteomic levels. (C) ROC curves of proteins (AUC > 0.7) with 5‐year OS. (D) Forest plot for the ten selected proteins (univariate Cox regression analysis). (E) Boxplot of NNMT mRNA expression in tumor and normal tissues based on the TCGA‐ccRCC dataset (created with GEPIA2). (F) Boxplot of NNMT protein expression in normal and tumor tissues from the CPTAC‐ccRCC dataset. (G) NNMT protein expression in normal and early‐stage (TNM stage I–II) tumor tissues. (H) NNMT protein expression stratified by pathological grade. (I) NNMT protein expression across different tumor stages. (J) Kaplan–Meier OS curve for NNMT expression in CPTAC‐ccRCC patients. (K) ROC curve of NNMT protein for predicting 5‐year OS. (L) UMAP plot showing cell type annotations after clustering in single‐cell RNA‐seq data (GSE171306). (M) Expression pattern of NNMT across different cell types in single‐cell RNA‐seq data. Significance was assessed using the Wilcoxon test (E–G), while comparisons across tumor grades and stages were performed with the Kruskal–Wallis test (H, I). Kaplan–Meier survival analyses were conducted using the log‐rank test (J). Graphs were generated using the ggplot2 package. (***p < 0.001). GO, Gene Ontology; DEGs, differentially expressed genes; DEPs, differentially expressed proteins; OS, overall survival; ROC, receiver operating characteristic; AUC, area under curve; UMAP, uniform manifold approximation and projection.

To identify robust prognostic biomarkers in ccRCC, we conducted a comprehensive analysis combining transcriptomic and proteomic data from public datasets (Figure 1A; Figure S1A). Candidate proteins that were consistently associated with mRNA levels and prognosis were subjected to Gene Ontology (GO) enrichment analysis. GO analysis revealed significant associations with ECM remodeling, collagen fibril structures, and the collagen‐containing matrix (Figure 1B). Among the consistently regulated and risk‐associated proteins, ten candidate biomarkers demonstrated strong predictive power for 5‐year overall survival (OS), as shown by receiver operating characteristic (ROC) analysis with an area under the curve (AUC) >0.7 (Figure 1C). NNMT, with the greatest prognostic performance, was selected for further evaluation. Univariate Cox regression analysis revealed that these proteins had significant hazard ratios with narrow confidence intervals, highlighting their potential as risk‐associated factors (Figure 1D). We next investigated the expression pattern of NNMT at both the proteomic and transcriptomic levels. NNMT mRNA levels were markedly higher in tumor tissues than in adjacent normal tissues (Figure 1E). Protein expression data from the Clinical Proteomic Tumor Analysis Consortium (CPTAC) cohort confirmed that NNMT expression was significantly higher in ccRCC patient tumors than in normal renal tissues (Figure 1F). Importantly, stratification by pathological stage revealed that even at early stages (TNM stages I–II), NNMT protein expression was already significantly upregulated (*p* < 0.001), suggesting its potential involvement in tumor initiation and its value as an early diagnostic marker (Figure 1G). Further stratification by histological grade and tumor stage revealed a progressive increase in NNMT protein expression with increasing pathological grade (Figure 1H) and American Joint Committee on Cancer (AJCC) TNM stage (Figure 1I), suggesting that NNMT expression is positively correlated with disease progression. Survival analysis based on the CPTAC cohort revealed that high NNMT expression corresponded to unfavorable OS (log‐rank *p* < 0.01; Figure 1J). Consistent with these findings, the ROC curve showed that the NNMT protein was a robust predictor of 5‐year OS (AUC = 0.789) (Figure 1K), further supporting its potential as a good prognostic biomarker. Subsequently, to explore the cellular origin of NNMT expression, we analyzed a single‐cell RNA‐seq dataset of ccRCC samples. Clustering and uniform manifold approximation and projection (UMAP) visualization identified tumor and stromal cell populations (Figure 1L). NNMT expression was predominantly enriched in epithelial tumor cells (Figure 1M). Overall, these findings highlight NNMT as a metabolically linked, early‐upregulated, and pathological stage‐relevant biomarker that may contribute to tumor progression and poor outcomes in patients with ccRCC. To further characterize NNMT in ccRCC, immunofluorescence staining was performed to assess its subcellular localization. As shown in Figure S1B, NNMT protein was localized mainly in the cytoplasm and nucleus of tumor cells, which is consistent with its role as a cytosolic enzyme involved in NAM methylation. Serum NNMT levels were measured by enzyme‐linked immunosorbent assay (ELISA) in an independent cohort of patients with ccRCC and age‐ and sex‐matched healthy controls. NNMT was either extremely low or undetectable in serum, indicating that it is unlikely to function as a secretory protein (Figure S1C,D).

### 2.2 NNMT Promotes Proliferation, Invasion, and Migration in ccRCC

**FIGURE 2:**
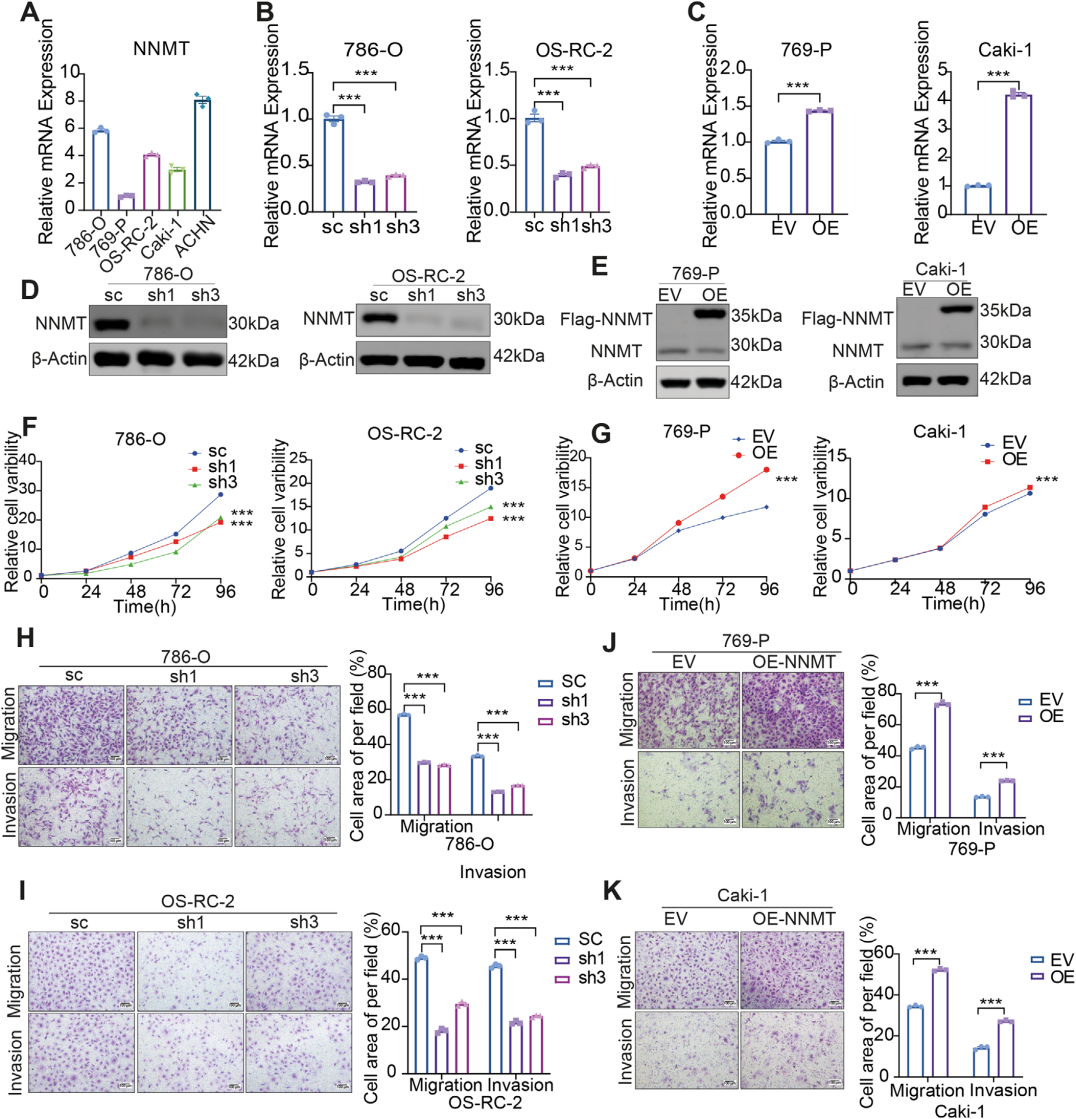
NNMT promotes proliferation, invasion, and migration in ccRCC cells. (A) NNMT mRNA expression levels in different ccRCC cell lines (relative to the lowest expression in 769‐P cells). (B, C) Validation of NNMT knockdown (B) and overexpression (C) at the mRNA level. (D, E) Validation of NNMT knockdown (D) and overexpression (E) at the protein level. (F, G) Cell proliferation assays following NNMT knockdown (F) and overexpression (G). (H–K) Transwell assays assessing migration and invasion abilities after NNMT knockdown (H, I) and overexpression (J, K). Significance was assessed using one‐way ANOVA followed by Dunnett's multiple comparisons test (B, F, H, I) or Student's *t*‐test (C, G, J, K). (***p < 0.001). EV, empty vector; OE, overexpression.

To investigate the functional role of NNMT in ccRCC, we first examined its basal expression across five ccRCC cell lines. NNMT expression levels varied among the different cell lines, with relatively high levels observed in OS‐RC‐2 and 786‐O cells, and low levels in Caki‐1 and 769‐P cells (Figure 2A). To manipulate NNMT expression, both knockdown and overexpression models were constructed. Quantitative real‐time PCR (RT‐qPCR) confirmed the efficient knockdown of NNMT in OS‐RC‐2 and 786‐O (Figure 2B), whereas NNMT overexpression was successfully established in Caki‐1 and 769‐P (Figure 2C). Western blotting analysis further validated the corresponding alterations in NNMT protein levels (Figure 2D,E). Cell Counting Kit‐8 (CCK‐8) assays revealed a significant decrease or increase in the growth rates of NNMT‐silenced (786‐O and OS‐RC‐2) and NNMT‐overexpressing (769‐P and Caki‐1) cells compared with their respective controls (Figure 2F,G). Migration and invasion assays demonstrated the functional role of NNMT in regulating cell motility. NNMT knockdown markedly impaired the migratory and invasive abilities of OS‐RC‐2 and 786‐O (Figure 2H,I), whereas NNMT overexpression enhanced these phenotypes in Caki‐1 and 769‐P (Figure 2J,K). Consistent results were obtained with small interfering RNA (siRNA) mediated NNMT silencing in 786‐O and ACHN (Figure S2A–H). Taken together, these results suggest that NNMT promotes ccRCC cell proliferation and motility.

### 2.3 NNMT Promotes Tumor Progression Independently of Its Substrates NAM and Products MNAM

**FIGURE 3:**
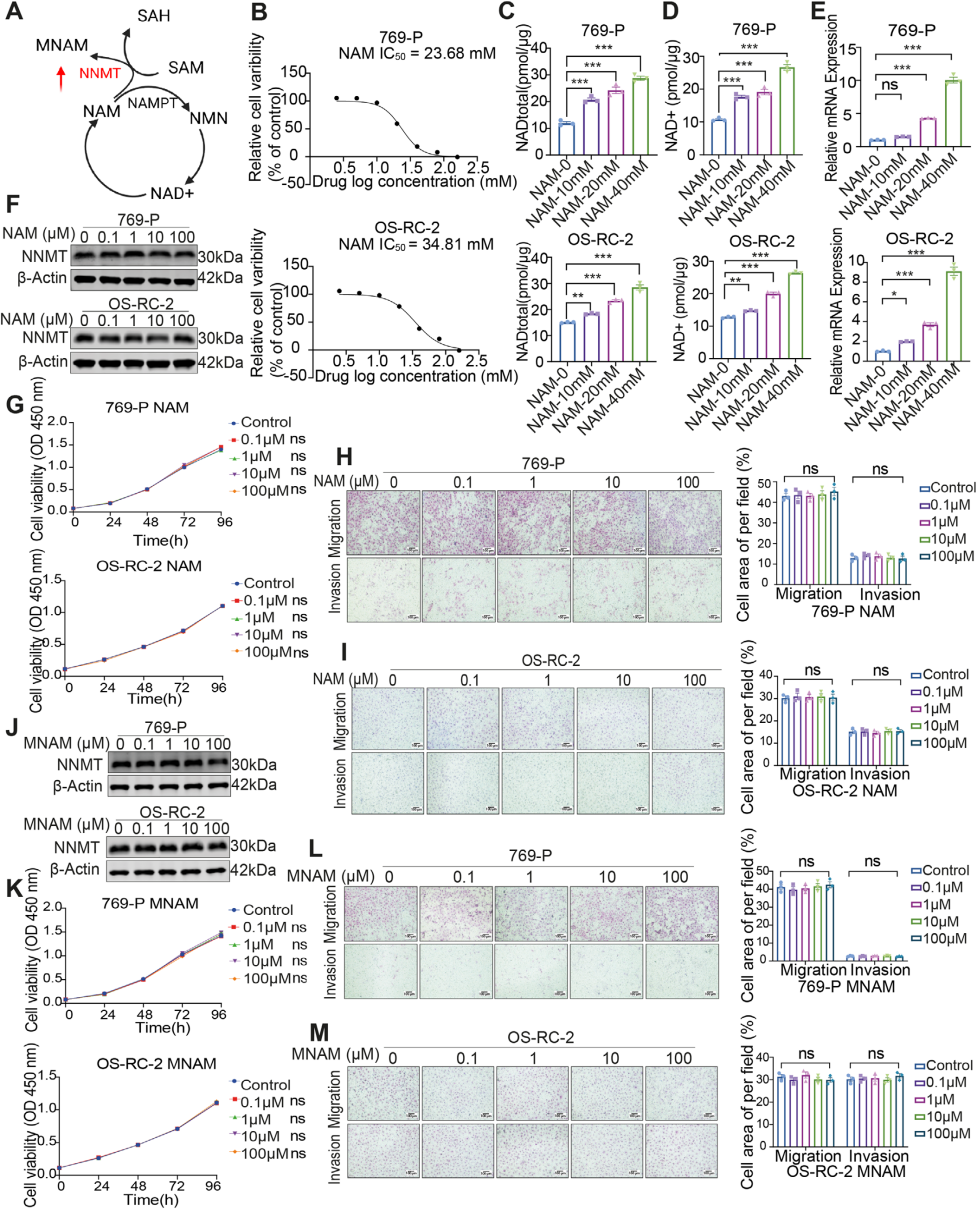
NNMT metabolic substrate NAM and product MNAM failed to promote tumor progression. (A) Schematic diagram of the NAM metabolism pathway. (B) IC_50_ values of NAM, (C) total NAD, and (D) NAD^+^ levels in 769‐P and OS‐RC‐2 cells after NAM treatment. (E) NNMT mRNA expression and (F) NNMT protein expression in 769‐P and OS‐RC‐2 cells following NAM treatment for 96 h. (G) Proliferation of 769‐P and OS‐RC‐2 cells after NAM treatment. (H, I) Migration and invasion assays of (H) 769‐P and (I) OS‐RC‐2 cells following NAM treatment for 96 h. Quantification of the areas of migrated and invaded cells on the lower surface of transwell chambers after NAM treatment. (J) NNMT protein expression in 769‐P and OS‐RC‐2 cells following MNAM treatment. (K) proliferation, (L, M) migration and invasion assays of 769‐P and OS‐RC‐2 cells following MNAM treatment for 96 h. Significance was assessed using one‐way ANOVA followed by Dunnett's multiple comparisons test. (*p < 0.05; **p < 0.01; ***p < 0.001; ns, not significant). NAM, nicotinamide; MNAM, 1‐methylnicotinamide; IC_50_, half maximal inhibitory concentration.

NNMT is involved in the NAM metabolism pathway, where it functions in the methylation of NAM to 1‐methylnicotinamide (MNAM), potentially altering NAD^+^ availability and redox balance within the tumor, and reducing the intracellular levels of SAM through the consumption of methyl donors (Figure 3A). To determine the effect of NAM, a precursor in the NAD^+^ salvage pathway, on ccRCC cells, we measured the median inhibition concentration (IC_50_) of NAM in 769‐P and OS‐RC‐2 cells (Figure 3B) and established concentration gradients surrounding the IC_50_ values. Subsequent analysis of total intracellular NAD (NAD^+^ + NADH) and NAD^+^ levels revealed that NAM treatment led to a marked production in the levels of both metabolites (Figure 3C,D; Figure S3A–C), suggesting that NAM indeed perturbs NAD metabolism via the cycle pathway illustrated in Figure 3A. To elucidate the mechanism, we examined NNMT expression following NAM treatment. Although NAM treatment led to the upregulation of NNMT mRNA levels (Figure 3E; Figure S2D), it did not affect NNMT protein levels across different concentrations and treatment durations (Figure 3F; Figure S3E). Moreover, silencing NNMT with siRNA led to a corresponding reduction in NAD levels (Figure S3F,G). Functionally, Cell proliferation assays revealed that continuous NAM exposure did not affect cell proliferation (Figure 3G). Transwell experiments demonstrated that NAM treatment did not change the migration or invasion capacities of ccRCC cells (Figure 3H,I; Figure S3H,I). Given that NAM can be methylated by NNMT to generate its methylated metabolite MNAM, we further evaluated the effects of MNAM on ccRCC cells. The IC_50_ of MNAM was shown in Figure S3J. MNAM did not significantly affect NNMT protein expression (Figure 3J). Furthermore, proliferation assays revealed that MNAM did not significantly promote ccRCC cell growth (Figure 3K) and exerted no significant effect on migration or invasion (Figure 3L,M). Similar to NAM supplementation, MNAM supplementation did not directly affect tumor malignant behavior. We conducted experiments using extended concentration ranges of the drugs and obtained consistent results (Figure S3K,L). Collectively, these findings suggest that NAM and its metabolite MNAM perturb NAD^+^ homeostasis but do not alter NNMT protein expression or affect the proliferation, motility, or invasive capacity of ccRCC cells, indicating that the NNMT substrate NAM and its metabolite MNAM are unlikely to directly drive ccRCC progression.

### 2.4 NNMT Promotes FN1 Expression Through H3K9me3

**FIGURE 4:**
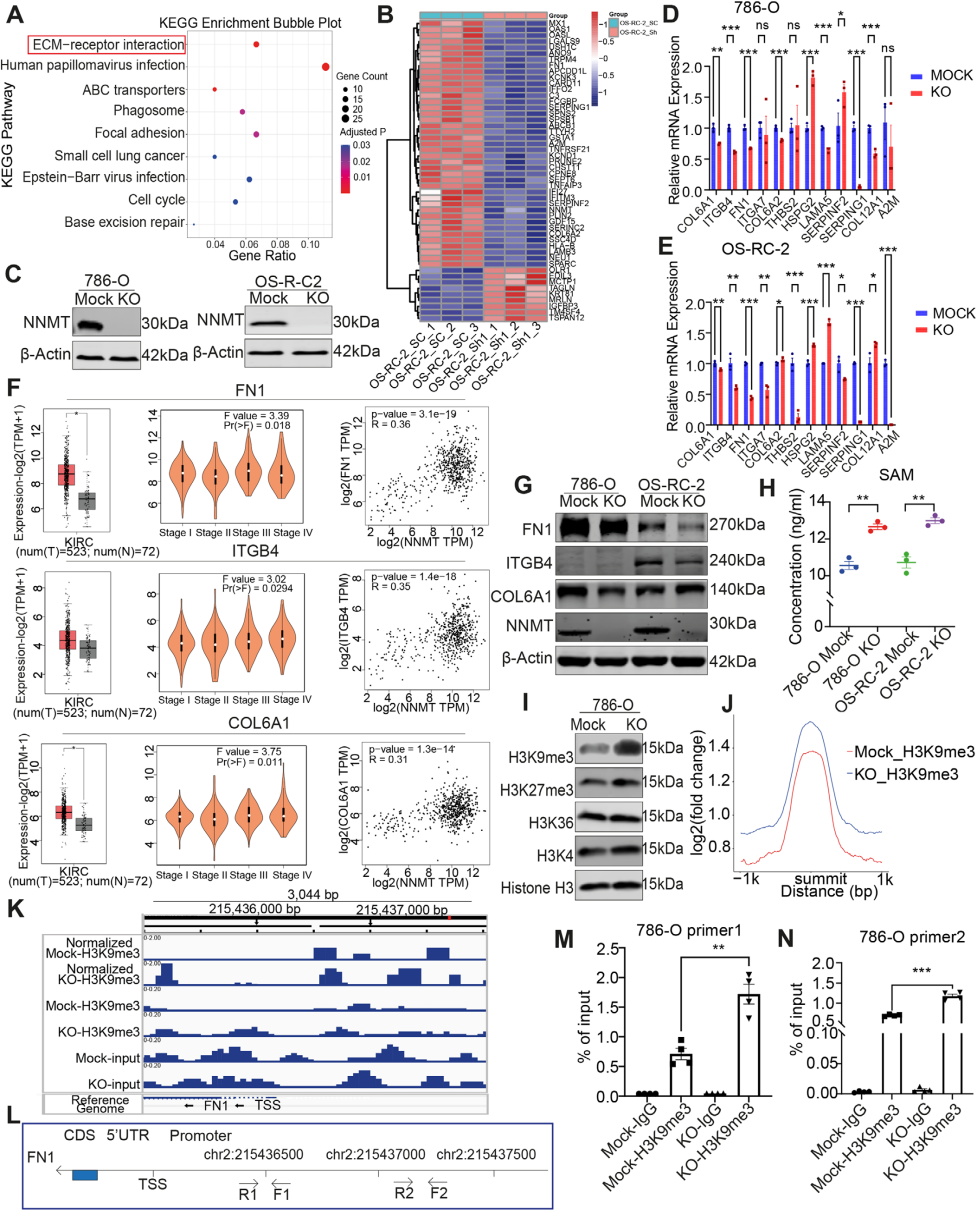
NNMT–H3K9me3 regulatory axis promotes FN1 expression. (A) KEGG pathway enrichment analysis of DEGs between OS‐RC‐2 control (OS‐RC‐2 SC) and NNMT‐knockdown (OS‐RC‐2 shNNMT) cells. (B) Heatmap showing the top 50 DEGs. (C) Protein‐level validation of NNMT‐KO in 786‐O and OS‐RC‐2 cells. (D, E) RT‐qPCR validation of ECM signaling‐related genes. (F) Box plots of mRNA expression levels, stage‐related expression, and correlation with NNMT for COL6A1, FN1, and ITGB4 (created with GEPIA2). (G) Western blotting validation of COL6A1, FN1, and ITGB4 expression changes in NNMT‐KO cells. (H) Measurement of intracellular SAM levels in Mock and NNMT‐KO cells. (I) Alterations in histone methylation patterns in 786‐O NNMT‐KO cells. (J, K) ChIP‐seq analysis of global H3K9me3 levels in regions adjacent to summits (J) and H3K9me3 enrichment at the FN1 promoter (K) in OS‐RC‐2 Mock and NNMT‐KO cells. (L) Schematic of two primer sites in the FN1 promoter region. (M, N) ChIP‐qPCR analysis of H3K9me3 enrichment at the FN1 promoter region. Significance was assessed using Student's *t*‐test (H, M, N), multiple unpaired *t*‐tests followed by two‐stage step‐up FDR correction (Benjamini, Krieger, and Yekutieli) (D, E), and the Kruskal–Wallis test for the tumor stage and Wilcoxon test for the expression level (F). (*p < 0.05; **p < 0.01; ***p < 0.001; ns, not significant). ChIP‐seq, chromatin immunoprecipitation sequencing; DEGs, differentially expressed genes; SAM, S‐adenosylmethionine.

To elucidate the molecular mechanism through which NNMT regulates transcriptional processes and promotes tumor progression in ccRCC, we performed differential expression analysis of transcriptomic data from NNMT‐knockdown OS‐RC‐2 cells and control cells. Kyoto Encyclopedia of Genes and Genomes (KEGG) pathway enrichment analysis revealed significant downregulation of ECM‐related signaling pathways, particularly ECM‐receptor interactions and cell adhesion molecules (Figure 4A). These findings suggest that NNMT promotes tumor invasiveness by modulating ECM components. These transcriptomic findings are consistent with our previous bioinformatics analysis of The Cancer Genome Atlas‐ccRCC (TCGA‐ccRCC) and CPTAC‐ccRCC patient datasets, where Gene Ontology (GO) enrichment also indicated significant alterations in ECM‐related biological processes (Figure 1B). Heatmap analysis revealed marked downregulation of ECM‐related genes in NNMT‐knockdown cells (Figure 4B). To investigate the mechanism underlying this regulation, we constructed and validated NNMT knockout (NNMT‐KO) cells (Figure 4C). Consistently, mRNA expression levels of fibronectin 1 (FN1), integrin subunit beta 4 (ITGB4), and collagen type VI alpha 1 chain (COL6A1) were obviously reduced in NNMT‐KO cells compared with Mock (control) cells (Figure 4D,E). Analysis of TCGA datasets revealed that FN1, ITGB4, and COL6A1 mRNA levels were upregulated in tumors, increased with tumor stage, and positively correlated with NNMT expression (Figure 4F). Survival analysis suggested that high expression levels of several ECM‐related genes validated in our study exhibited a tendency toward poorer prognosis in patients with ccRCC (Figure S4A). Given the most prominent protein‐level change, specifically FN1 (Figure 4G), we subsequently investigated the signaling pathways underlying NNMT regulation.

Considering the intrinsic function of NNMT as a methyltransferase, we detected increased SAM levels in NNMT‐KO cells of both 786‐O and OS‐RC‐2 cell lines, indicating that ccRCC Mock cells with proficient NNMT expression exhibited relatively lower SAM levels (Figure 4H). Therefore, we examined the impact of NNMT‐KO on histone methylation modifications. Among the various histone marks tested upon NNMT‐KO cells, H3K9me3, a canonical heterochromatin marker, exhibited the most notable increase, suggesting a potential epigenetic mechanism for NNMT‐mediated regulation of gene transcription (Figure 4I; Figure S4B). Following these observations, chromatin immunoprecipitation sequencing (ChIP‐seq) analysis was performed in NNMT‐KO and Mock cells to evaluate H3K9me3 levels. The results showed that NNMT‐KO led to a global increase in H3K9me3 (Figure 4J) and enhanced enrichment of H3K9me3 at the FN1 promoter region relative to Mock cells (Figure 4K). ChIP‒qPCR primers targeting the FN1 promoter (Figure 4L) were used to validate the potential epigenetic regulation. ChIP‒qPCR revealed a significant enrichment of H3K9me3 at the FN1 promoter after NNMT knockout (Figure 4M,N; Figure S4C). Supplementation with S‐adenosyl‐L‐methionine disulfate tosylate (SAMe) as a methyl donor further increased H3K9me3 modification and reduced the expression level of FN1 (Figure S4D). These results indicate that NNMT modulates FN1 transcription by influencing local histone methylation status. Specifically, NNMT promotes the expression of the ECM‐related gene FN1 by reducing H3K9me3‐mediated transcriptional repression at its promoter, thereby activating tumor microenvironment signaling and facilitating invasive behavior in ccRCC cells. These findings reveal a novel epigenetic mechanism of NNMT in tumor progression.

Additionally, based on the RNA‐seq results in OS‐RC‐2 cells, we selected collagen type I alpha 1 chain (COL1A1), a gene whose expression was unaffected by NNMT, as a negative control. Two primer pairs were designed near the promoter region for ChIP‐qPCR analysis, with no statistically significant enrichment observed (Figure S4E,F). To determine whether NNMT regulates methylation processes beyond histone methylation, coimmunoprecipitation (Co‐IP) assays were performed in HEK293T cells that overexpress NNMT to assess its direct interaction with key proteins involved in m6A RNA methylation (Figure S4G). The results showed no stable interactions between NNMT and m6A regulatory factors, suggesting that the epigenetic regulatory mechanism of NNMT is unlikely to involve direct modulation of the m6A pathway. Meanwhile, examination of H3K9 methyltransferases did not identify any molecules with consistently altered expression in either cell line (Figure S4H,I).

Given that FN1 is a key component of the ECM and is closely associated with epithelial–mesenchymal transition (EMT), we performed gene set enrichment analysis (GSEA) on transcriptomic data from OS‐RC‐2 cells to determine whether EMT‐related pathways were altered. GSEA revealed enrichment of several oncogenic pathways, including EMT, hypoxia‐related, and PI3K‐AKT pathways, which are activated in cells with relatively high NNMT expression (Figure S4J). To further validate these findings, we examined the expression of key EMT markers. NNMT‐KO promoted E‐cadherin expression and concurrently inhibited ZEB1 and N‐cadherin (Figure S4K). We also detected that the levels of pFAK and pPI3K, both downstream effectors of FN1, were significantly decreased (Figure S4L). These observations reveal that NNMT contributes to the migratory and invasive behavior of ccRCC cells partly through the regulation of ECM remodeling and EMT‐associated gene expression.

### 2.5 FN1 Restoration Confirms the Role of the NNMT–FN1 Axis in Driving Tumor Metastasis

**FIGURE 5:**
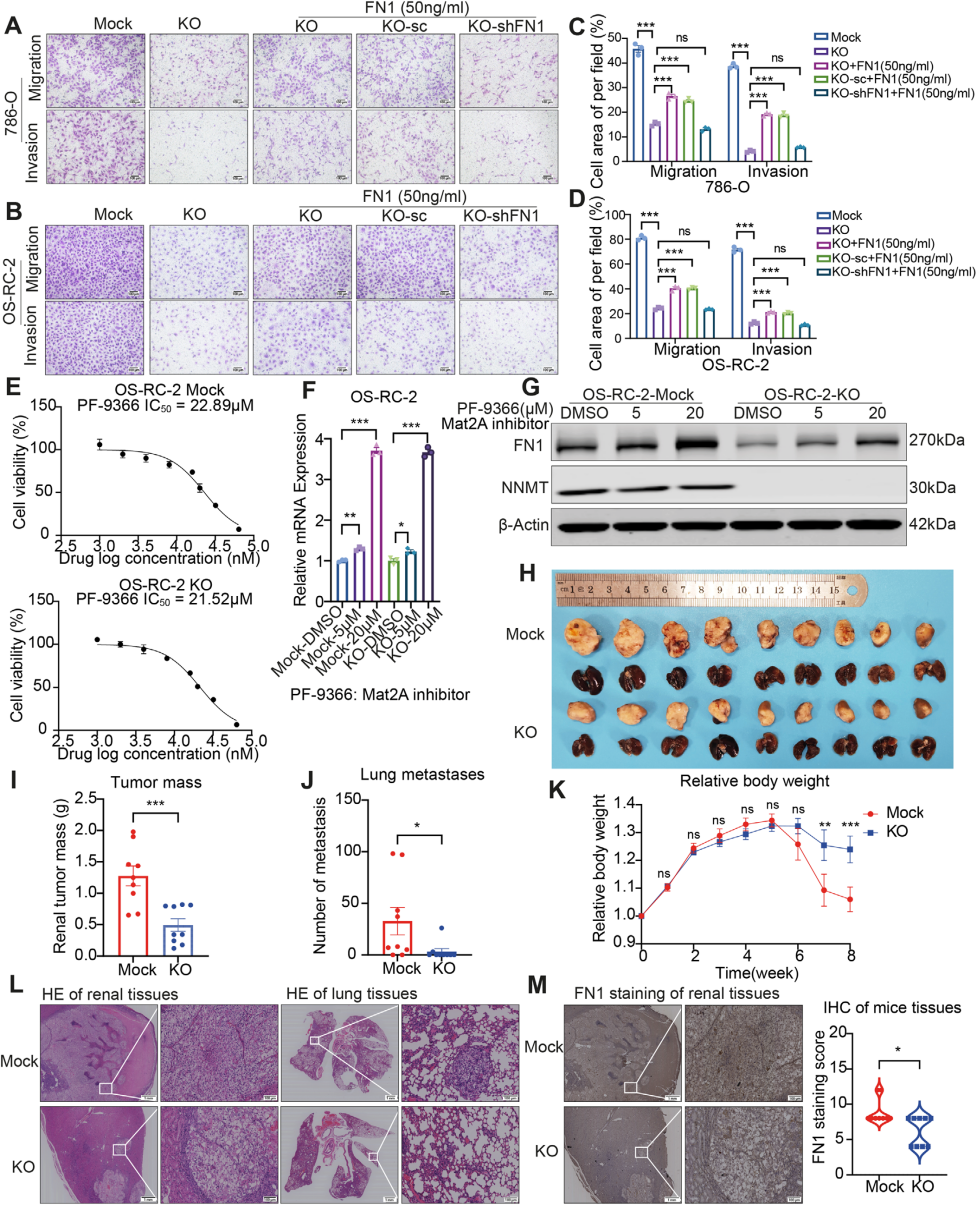
FN1 rescues NNMT‐driven tumor progression. (A, B) Effects of exogenous recombinant FN1 protein and shFN1 on the migration and invasion. (C, D) Quantification of migrated and invaded cells. (E) IC_50_ values of the PF‐9366 in OS‐RC‐2 Mock and NNMT‐KO cells. (F) FN1 mRNA levels in OS‐RC‐2 cells treated with PF‐9366. (G) FN1 protein levels in OS‐RC‐2 cells after PF‐9366 treatment. (H) Effects of NNMT‐KO in OS‐RC‐2 cells on tumor growth (*n* = 9 per group), (I) tumor mass (*n* = 9 per group, tumor mass = mass of tumor‐bearing renal − mass of contralateral renal), (J) number of lung metastases (*n* = 9 per group), and (K) relative body weight of mice (*n* = 9 per group). (L) Representative H&E staining of renal and lung tissues. (M) Representative FN1 staining of renal tissues by IHC (left panel), and quantification of the immunoreactive score (right panel, *n* = 9 per group). Significance was assessed using one‐way ANOVA followed by Dunnett's multiple comparisons test (C, D, F), two‐way ANOVA followed by Sidak's multiple comparisons test (K), or Student's *t*‐test (I, J, M). (*p < 0.05; **p < 0.01; ***p < 0.001; ns, not significant). H&E, hematoxylin and eosin; IHC, immunohistochemistry; PF‐9366, methionine adenosyltransferase 2A (Mat2A) inhibitor (HY‐107778, MCE).

To elucidate whether FN1 acts downstream of NNMT to mediate tumor progression, we conducted a series of rescue experiments. FN1 was knocked down in 786‐O and OS‐RC‐2 cells, including Mock and NNMT‐KO groups (Figure S5A,B), and subsequent cell migration and invasion were evaluated. The results showed that FN1 knockdown reduced migration and invasion in both Mock and NNMT‐KO cells (Figure S5C,D). Exogenous supplementation with recombinant FN1 protein significantly increased cell migration and invasion in Transwell assays (Figure S5E,F), whereas additional FN1 knockdown partially attenuated these rescue effects (Figure 5A–D), indicating that FN1 can effectively restore the migration and invasion abilities impaired by NNMT knockout. To further validate FN1 as a downstream target of NNMT, we measured the IC_50_ of PF‐9366 to guide subsequent dosing. PF‐9366 is a methionine adenosyltransferase 2A (Mat2A) inhibitor that inhibits SAM synthesis, thereby simulating the methyl consumption caused by NNMT in NNMT‐KO and Mock control cells (Figure 5E). Treatment with PF‐9366 resulted in a pronounced increase in FN1 mRNA expression (Figure 5F) and protein levels (Figure 5G), confirming that FN1 is a downstream target of NNMT‐mediated signaling. To assess the in vivo relevance, orthotopic tumor models were established using OS‐RC‐2 cells. Mice implanted with NNMT‐KO cells developed markedly smaller tumor volume and tumor mass, as well as fewer lung metastases, compared with controls (Figure 5H–J). In addition, tumor‐induced cachexia was alleviated (Figure 5K). Orthotopic tumors and lung metastases were further confirmed by histopathological examination via hematoxylin and eosin (H&E) staining (Figure 5L). Immunohistochemistry (IHC) revealed that compared with that in the control group, FN1 expression in renal tumors from the NNMT‐KO group was substantially lower (Figure 5M). Together, these results demonstrate that NNMT promotes ccRCC progression and metastasis by upregulating FN1 expression, which enhances tumor cell migration and invasion. Importantly, FN1 supplementation partially rescued the attenuated malignant phenotypes caused by NNMT depletion, suggesting that targeting the NNMT‐FN1 axis may provide therapeutic benefit in ccRCC.

### 2.6 NNMT Highly Positively Correlated With FN1 as a Potential Therapeutic Target

**FIGURE 6:**
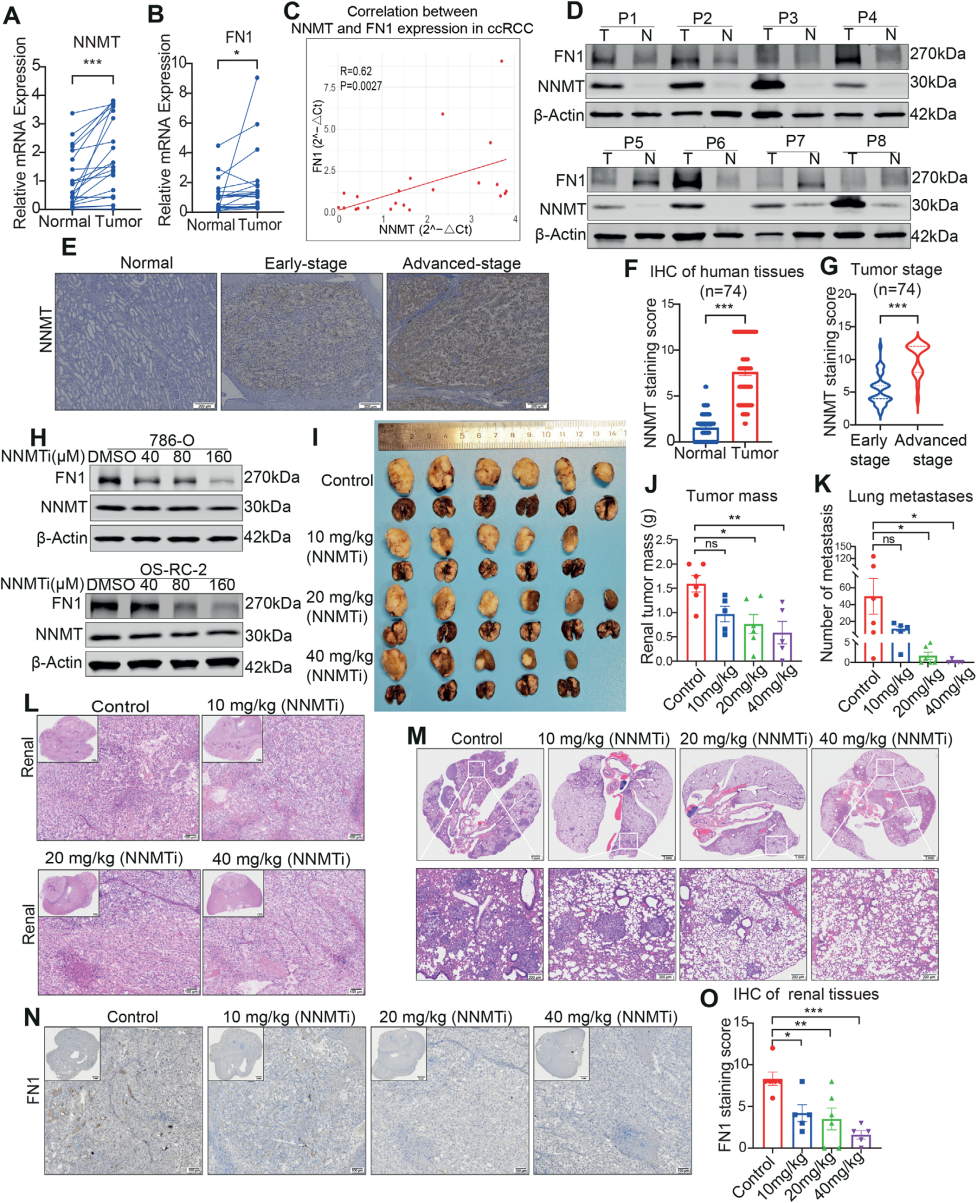
Upregulated NNMT and FN1 in ccRCC with therapeutic potential of NNMT inhibitor. (A, B) NNMT and FN1 mRNA expression in paired ccRCC patients (*n* = 22) (relative levels were presented using the 2^−ΔCt^ method). (C) NNMT and FN1 correlation in tumor tissues. (D) Western blotting analysis of NNMT and FN1 protein expression in paired ccRCC patients. (E–G) Representative IHC images (E) and quantification scores of NNMT staining in ccRCC patient tissues (*n* = 74). (F) Comparison between normal and tumor. (G) Comparison between early‐stage and advanced‐stage. (H) NNMTi treatment in OS‐RC‐2 and 786‐O cells for 96 h. (I–K) Effects of NNMTi in OS‐RC‐2 cells on (I) tumor growth (control, *n* = 6; 10 mg/kg, *n* = 5; 20 mg/kg, *n* = 6; 40 mg/kg, *n* = 5), (J) tumor mass (control, *n* = 6; 10 mg/kg, *n* = 5; 20 mg/kg, *n* = 6; 40 mg/kg, *n* = 5, tumor mass **=** mass of tumor‐bearing renal − mass of contralateral renal), (K) number of lung metastases (control, *n* = 6; 10 mg/kg, *n* = 5; 20 mg/kg, *n* = 6; 40 mg/kg, *n* = 5). Representative H&E staining of (L) renal and (M) lung tissues. (N) Representative FN1 staining of renal tissues by IHC. (O) Quantification of IHC score for renal tissues (control, *n* = 6; 10 mg/kg, *n* = 5; 20 mg/kg, *n* = 6; 40 mg/kg, *n* = 5). Significance was assessed using the Wilcoxon test (A, B, F, G) or one‐way ANOVA (J, K, O) followed by Dunnett's multiple comparisons test. (*p < 0.05; **p < 0.01; ***p < 0.001; ns, not significant). H&E, hematoxylin and eosin; IHC, immunohistochemistry; NNMTi, NNMT inhibitor (HY‐131042, MCE).

Examination of freshly collected adjacent normal tissues and paired tumor samples revealed a significant upregulation of NNMT and FN1 transcription levels in tumor tissues (Figure 6A,B). Furthermore, a strong positive correlation between NNMT and FN1 mRNA expression was observed in tumor samples (Spearman correlation *R* = 0.62, *p* = 0.0027 in tumor tissues; Figure 6C). We validated this correlation using public datasets: GSE46699 (130 samples, 65 paired cases) and GSE53757 (144 samples, 72 paired cases). Both datasets were consistent with our cohort, particularly the GSE46699 dataset, which showed a correlation of *R* = 0.527 and *p* < 0.001 (Figure S6A). In addition, the TCGA‐ccRCC dataset also confirmed a positive correlation (*R* = 0.36, *p* < 0.001), as shown in Figure 4F. Moreover, the upregulation of NNMT in tumors was validated using the European Gene Expression Omnibus (GEO) datasets (Figure S6B).

Concordantly, NNMT protein expression (Figure 6D–F) were substantially elevated in tumor specimens compared with adjacent normal tissues, and were associated with tumor stage (*n* = 74; Figure 6G; Figure S6C). The comparison of clinicopathological characteristics between the NNMT high‐expression and low‐expression groups of patients with ccRCC is shown in Table S1. Western blotting demonstrated that treatment with an NNMT inhibitor (NNMTi) profoundly suppressed FN1 protein expression in cells (Figure 6H). In vivo studies demonstrated that NNMTi treatment dose‐dependently reduced orthotopic tumor size (Figure 6I), tumor mass (Figure 6J), and the number of lung metastases, as determined by H&E staining (Figure 6K). Representative H&E staining results of renal and lung tissues are shown in Figure 6L,M. IHC analysis revealed that FN1 expression in mouse renal tumors was downregulated following NNMTi treatment, exhibiting a dose‐dependent effect (Figure 6N,O). To investigate downstream signaling linked to FN1 downregulation in vivo, IHC was performed to assess pPI3K levels in tumors from Mock and NNMT‐KO xenograft mice, as well as in mice treated with NNMTi. Compared with those in the control group, pPI3K levels in the experimental groups were downregulated (Figure S6D–G). Taken together, these results support the notion that NNMT may represent a promising therapeutic target.

## 3 Discussion

Although early‐stage ccRCC is generally curable, therapeutic options for metastatic disease remain limited [28] and pose a significant health burden. The role of NNMT, a methyltransferase implicated in tumorigenesis, in driving ccRCC progression remains largely unexplored [29]. Evidence from this work suggests that elevated NNMT expression is associated with poor prognosis in patients with ccRCC by analyzing multi‐omics datasets and investigating the impact of NNMT in ccRCC. NNMT regulates FN1 expression through H3K9me3‐dependent epigenetic mechanisms, providing novel insights into the metabolic‐epigenetic crosstalk that drives ECM gene expression and tumor aggressiveness. Targeting NNMT may disrupt tumor‐promoting pathways mediated by FN1, offering a new approach for preventing tumor invasion and metastasis. Among various tumor types, ccRCC is particularly closely linked to metabolic processes [15, 16, 17]. Within the NNMT‐mediated metabolic pathway, NAM serves as the substrate, MNAM as the product, and SAM as the methyl donor consumed during the reaction. These metabolic features may underlie the functional role of NNMT in renal tumor progression. MNAM is an inert metabolite that is excreted in urine [11]. In our study, we found that the addition of NAM or MNAM to tumor cells did not significantly affect their malignant capacity. These findings were consistent with previous research showing that MNAM treatment also failed to increase the migratory ability of tumor cells, which is in line with previous observations in other cancer models [1]. Furthermore, no significant differences in serum MNAM levels were observed between patients with breast cancer, patients with benign tumors, and healthy controls [30]. These results suggest that, despite being a downstream metabolite of NNMT, MNAM may not play a direct role in promoting tumor growth or metastasis, at least in certain tumor contexts. However, previous findings [30, 31] have suggested that MNAM is produced by both cancer‐associated fibroblasts and cancer cells, indicating that MNAM is involved in tumor progression. Gujar et al. have demonstrated that NAD maintains cancer stemness in glioma cells [32], inhibits melanoma growth in vitro and in vivo [33], and promotes immune responses by upregulating PD‐L1 expression in hepatocellular carcinoma [34]. In our study, we also observed that alterations in NAD metabolism in ccRCC cells did not lead to noticeable changes in their migratory or invasive capacities. Our findings and published evidence suggest that enhanced NAM, MNAM, and NAD metabolism may promote tumor progression via mechanisms such as metabolic adaptation, stress resistance, or immune modulation, independent of tumor cell motility.

Transcriptomic analyses revealed that NNMT knockdown leads to the downregulation of multiple ECM‐related genes, particularly FN1, COL6A1, and ITGB4, which are pivotal mediators of metastatic dissemination [35, 36, 37]. We focused on FN1, whose expression changed most significantly at the protein level. Studies have reported that NNMT plays a critical role in promoting DNA damage repair in tumor cells, thereby contributing to radiotherapy resistance [11]. Regarding histone and RNA methylation, previous studies have reported that NNMT contributes to the invasive and metastatic behavior of hepatocellular carcinoma by modulating H3K27me3 and transcriptionally activating CD44, as well as facilitating the formation of the CD44v3 splice variant via NNMT‐mediated m6A modification of CD44 mRNA [38]. H3K9me3 is a well‐characterized epigenetic mark associated with transcriptional repression and heterochromatin formation. Recent studies have shown that H3K9me3 can regulate the expression of key transcription factors, including Snail and Myc, which are involved in tumor progression [39, 40]. In our study, ChIP‐seq and ChIP‐qPCR revealed increased enrichment of the repressive histone mark H3K9me3 at the FN1 promoter region following NNMT depletion, indicating that NNMT modulates ECM gene transcription through epigenetic remodeling.

ELISA assays revealed relatively low SAM levels in ccRCC cells with proficient NNMT expression, suggesting a potential role of NNMT in regulating intracellular methyl donor availability. This finding is supported by previous studies demonstrating that NNMT dysregulation significantly alters the cellular SAM pool across various tumor types, as evidenced by mass spectrometry analyses [3, 25]. Consistent with this mechanism, SAMe supplementation increased H3K9me3 levels and reduced FN1 protein expression, whereas disruption of cellular SAM biosynthesis by PF‐9366 produced the opposite effect. Restoration of FN1 in NNMT‐KO cells reversed migration and invasion, thereby confirming the functional relevance of the NNMT–H3K9me3–FN1 regulatory axis. In FN1‐mediated downstream pathways, the interaction between FN1 and its integrin receptors is a classical signaling pathway that drives tumor progression [41]. Notably, the PI3K‐AKT pathway was enriched in our GSEA analysis, which is consistent with previous reports showing that ECM‐mediated activation of EGFR phosphorylation, particularly through FN1, can subsequently trigger the EGFR/PI3K signaling cascade, promoting oncogenic processes [42, 43, 44]. Xenograft models demonstrated that NNMT‐KO suppresses tumor growth and lung metastasis. Furthermore, intraperitoneal injection of NNMTi in mice caused significant tumor regression in a dose‐dependent manner. Taken together, these findings establish NNMT as a central driver of ccRCC progression by linking metabolic and epigenetic mechanisms. Moreover, the antitumor effects of NNMTi are mediated through disruption of the NNMT–FN1 axis, highlighting its potential as a therapeutic target in ccRCC.

In terms of research limitations, the effects of NNMT‐mediated NAM metabolism, including metabolites such as SAM and NAD^+^, on the epigenome need to be comprehensively characterized across diverse cancer types. In addition, whether NNMT is functionally linked to m6A‐mediated pathways in ccRCC remains unclear, and the underlying regulatory mechanisms are complex and warrant further investigation. Future studies integrating genomic, transcriptomic, epigenomic, and metabolomic profiling will be essential for fully understanding the role of NNMT in ccRCC progression and therapeutic resistance.

## 4 Conclusions

**FIGURE 7:**
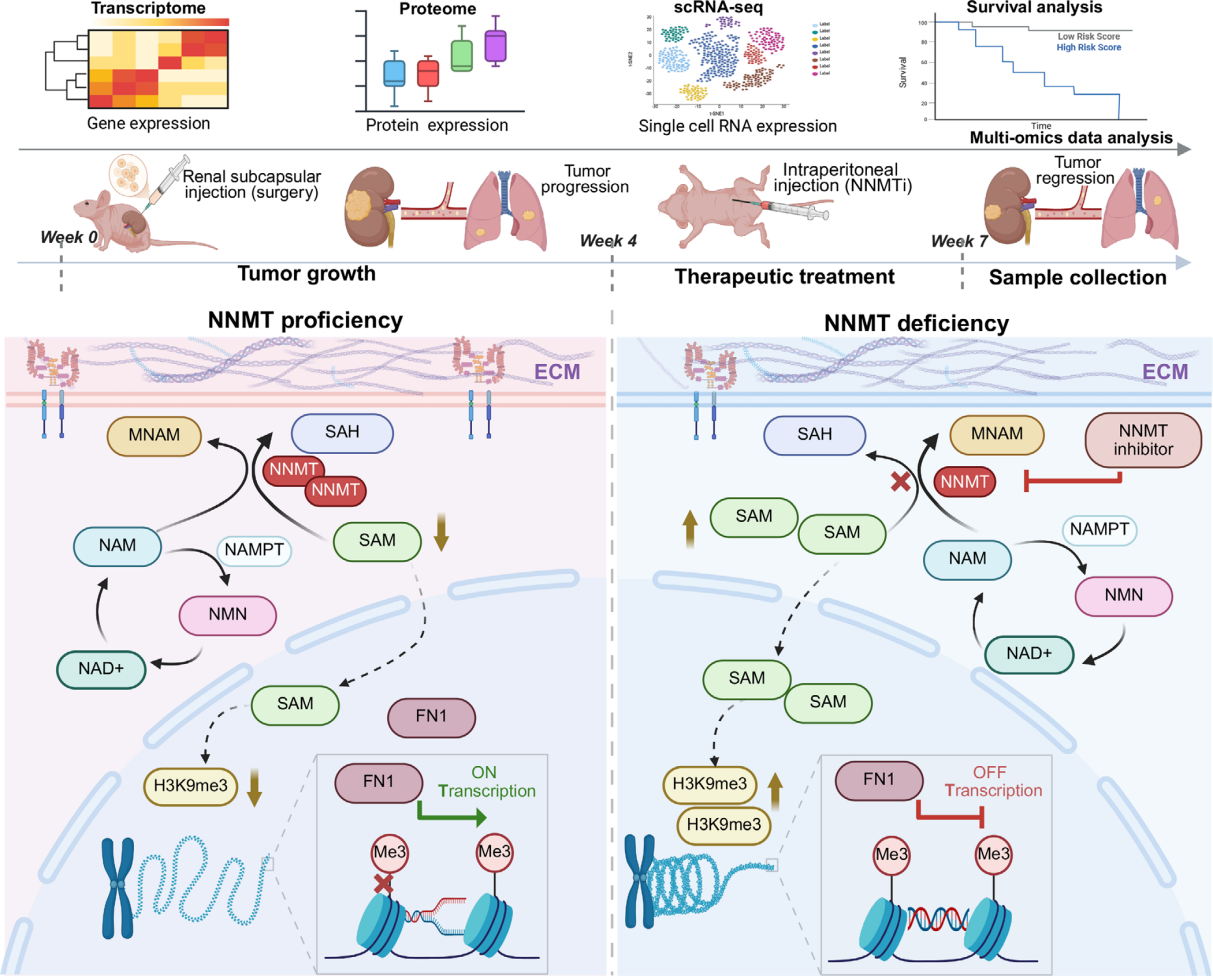
Summary of the NNMT–H3K9me3–FN1 axis in ccRCC. NNMT upregulation in ccRCC is associated with poor prognosis. Mechanistically, NNMT reduces SAM levels, thereby decreasing H3K9me3 modification and promoting FN1 transcription. This oncogenic pathway suggests that NNMT may serve as a promising biomarker for the development of precision therapies in ccRCC.

This study demonstrated that NNMT is upregulated in early‐stage disease and promotes ccRCC progression. Its effect was not mediated through its metabolic substrate NAM or product MNAM, but rather via a reduction in H3K9me3 at the FN1 promoter, leading to the upregulation of the key ECM component FN1. NNMT‐mediated epigenetic reprogramming enhances tumor cellular motility and invasiveness in vitro and in animal models. Moreover, NNMTi effectively suppressed FN1 expression in ccRCC. Taken together, these findings highlight the NNMT as a potential diagnostic biomarker and the NNMT‐FN1 axis as a promising therapeutic target for ccRCC (Figure 7).

## 5 Materials and Methods

### 5.1 Animals

The animal experiments passed the Animal Ethics Review [ZJU20240223], Zhejiang University. After the mouse tags were attached, the mice were randomly grouped completely (*n* = 9 per group). Six‐week‐old male nude mice received a renal subcapsular injection of 3 × 10^5^ OS‐RC‐2 cells (Mock or NNMT‐KO) suspended in PBS. Mouse body weights were recorded weekly. At the endpoint, tumors were excised, weighed, photographed, and subjected to histological analysis. Lung metastases were evaluated by counting metastatic nodules on H&E. Treatment assays were conducted four weeks after renal tumor implantation. Mice were then randomly grouped based on average fluorescence intensity (OS‐RC‐2 cells were transfected with the pGKV5 fluorescent plasmid) and treated with the vehicle control (*n* = 6) or NNMTi (MCE, HY‐131042; 10 mg/kg, *n* = 5; 20 mg/kg, *n* = 6; 40 mg/kg, *n* = 5) via intraperitoneal injection for 24 days. The inhibitor was prepared in a solvent consisting of 5% Tween 80 (Selleck, S6702) in combination with 40% PEG300 (Selleck, S6704) dissolved in 55% PBS, and the control group received the same vehicle. No mice were excluded after group allocation, and all animals were included in the final analysis.

### 5.2 Patient Samples

All the specimens were independently evaluated, and IHC scoring was performed by two certified pathologists. Tissue samples were taken from patients with ccRCC who had not received preoperative treatment, including radiotherapy, chemotherapy, or any other adjuvant treatments, before surgical resection. The Ethics Committee approved this study (No. 2021IIT923), First Affiliated Hospital at Zhejiang University.

### 5.3 Cell Lines

The ccRCC cell lines 786‐O, 769‐P, OS‐RC‐2, ACHN, and Caki‐1 were obtained from American Type Culture Collection (ATCC), and HEK293T cells were obtained from the Chinese Academy of Sciences. Penicillin and streptomycin (1% PS) and 10% fetal bovine serum (FBS) were added to RPMI‐1640 media for 786‐O, 769‐P, OS‐RC‐2, and ACHN cells, which were cultured at 37°C in a humidified incubator with 5% CO_2_. DMEM was used to culture both Caki‐1 and HEK293T cells under the same conditions. For cell constructions, cells were transfected with siRNAs (Sangon Biotech, China) targeting NNMT or the negative control (NC) using GenMute (SignaGen). Short hairpin RNA (shRNA) of NNMT and empty PLKO.1 transfections were performed using LipoD293 Transfection Reagent (SignaGen). For NNMT overexpression, NNMT cDNA or an empty PCDH plasmid was transfected into the cells with LipoD293.

### 5.4 Bioinformatics Analysis

Total RNA was extracted in triplicate from both NNMT‐knockdown and control OS‐RC‐2 cells, and sequenced by Novogene Co., Ltd. Differentially expressed genes (DEGs) were identified using the DESeq2 R package [45]. KEGG pathway enrichment was performed with the clusterProfiler R package [46], and GSEA was conducted using the GSEA software platform (https://www.broadinstitute.org/gsea/). All computational analyses for ccRCC were conducted using R software (version 4.0.2).

### 5.5 RT‐qPCR

RNA was extracted using the Trizol method (Pufei Biotechnology, 3101‐100). cDNA synthesis was performed with a reverse transcriptase kit (Vazyme, R223‐01). Assays were carried out by using SYBR Mix (Vazyme, Q711‐02) on a LightCycler 480 System. Relative levels were presented using the 2^−ΔΔCt^ method, though normalizing to β‐Actin.

### 5.6 Western Blotting

Cocktail (MCE, HY‐K0021, HY‐K0022, HY‐K0010) and RIPA buffer (Beyotime, P0013B) were added to the cells. For the MNAM (Sigma‐Aldrich, SML0704) and NAM (MCE, HY‐B0150) supplementary experiments, proteins were collected 96 h after drug treatment. NNMTi (MCE, HY‐131042) was applied to the cells for 96 h, and SAMe (MCE, HY‐W017770) and PF‐9366 (MCE, HY‐107778) for 72 h. After BCA (Thermo Scientific, 23225) and SDS‐PAGE assay, proteins were shifted onto nitrocellulose membranes. Protein bands were blocked in 5% nonfat milk or bovine serum albumin (BSA), and incubated with antibodies against NNMT (Santa Cruz, sc‐376048), Histone H3 (Proteintech, 17168‐1‐AP), FN1 (Proteintech, 66042‐1‐1g), COL6A1 (Proteintech, 17023‐1‐AP), ITGB4 (Proteintech, 21738‐1‐AP), H3K9me3 (Abcam, ab8898), H3K27me3 (HUABIO, HA722231), N‐Cadherin (CST, 13116), H3K4(HUABIO, HA722662), H3K36 (HUABIO, HA722802), ZEB1 (CST, 83243), E‐Cadherin (CST, 3195), Flag (Sigma, F1804‐200UG), HA (YoChe, AYD02‐100), Phospho‐FAK (CST, 3283S), FAK (Proteintech, 66258‐1‐1g), Phospho‐PI3K (Affinity, AF3242), Pl3K (Affinity, AF6241), and β‐Actin (Proteintech, 66009‐1‐Ig). After the membranes were incubated with fluorescent secondary antibodies, protein bands were analyzed using an Odyssey imaging system (LI‐COR). Histone H3 and histone methylation‐modifying proteins were in the same band positions. They may be incubated on different membranes.

### 5.7 NNMT‐KO Cells

sgRNA targeting NNMT was packaged in HEK293T cells and used to infect target ccRCC cells. Cells were selected with puromycin until all control cells were eliminated. Single cells were then isolated by flow cytometry into 96‐well plates and cultured for two weeks. Clonal expansion was monitored daily, and growing clones were subsequently expanded in 24‐well plates. Genomic DNA and protein were extracted for Sanger sequencing and western blotting to identify homozygous knockout clones.

### 5.8 NAD Quantification

NAD^+^ levels and total NAD were quantified using an NAD/NADH Assay Kit (Beyotime, S0175). Briefly, cell lysates were prepared, and NAD and NADH were extracted separately. A microplate reader was used to determine the concentrations (450 nm).

### 5.9 Proliferation and IC_50_ Assays

Cells (1 × 10^3^ for 786‐O, 2 × 10^3^ for OS‐RC‐2, 2 × 10^3^ for 769‐P, 2 × 10^3^ for Caki‐1, 3 × 10^3^ for ACHN, in the proliferation assays; 2 × 10^3^ cells for IC_50_ assays) grew in 96‐well plates and were treated as indicated. CCK‐8 (Biosharp, BS350C) was added to each well of a 96‐well plate, and the cells were measured after incubation (450 nm).

### 5.10 Migration and Invasion Assays

To promote cell adhesion, the underside of the Transwell membrane was coated with fibronectin (FN, Sigma‐Aldrich, F0895) and incubated at room temperature for 1–2 h before the cells were seeded. Transwell upper chambers (Corning, 3422) with (invasion assay) or without (migration assay) Matrigel (BD Biosciences, 356234) coating were used, respectively. Cells (1 × 10^4^ for 786‐O, 2 × 10^4^ for OS‐RC‐2, 2 × 10^4^ for 769‐P, 3 × 10^4^ for Caki‐1, 3 × 10^4^ for ACHN) were seeded in serum‐free medium (upper chamber). After 24–48 h, the cells on the Transwell membrane surface were collected, dyed, and quantified (ImageJ software, version 2).

### 5.11 ChIP‐qPCR and ChIP‐Seq

The ChIP assay was conducted using a SimpleChIP Kit (CST, #56383). Cells were crosslinked, chromatin was broken, and IP was performed with an anti‐H3K9me3 antibody (Abcam, ab8898) or an IgG control. Purified DNA was amplified with primers targeting the FN1 promoter region, and enrichment was calculated relative to the input DNA. Library construction and sequencing were performed by Sangon Biotech.

### 5.12 Hematoxylin and Eosin

Mouse tissues were initially fixed and subsequently processed for embedding. Tissue sections were cut, gradually deparaffinized, and gradually rehydrated through descending ethanol concentrations. Hematoxylin solution (Sigma) was applied for 10 min to stain nuclei, followed by a brief counterstaining with eosin (Sigma) for 2 s to visualize the cytoplasmic components. Digital images of the slides were acquired using a NanoZoomer slide scanner (Hamamatsu). Histological evaluation of tissue architecture and cellular features was conducted by experienced pathologists to identify morphological changes or abnormalities.

### 5.13 Immunohistochemistry

Paraffin‐embedded clinical tissue samples underwent dewaxing, hydration, and blocking of endogenous peroxidase activity. Then, antigen retrieval was performed in citrate or EDTA buffer (ZSGB‐BIO) for 3 min. After cooling, the samples were blocked with 10% bovine serum (Gibco) for around 30 min to reduce nonspecific reactions. Slides were incubated with the NNMT, FN1, or pPI3K primary antibody. Then, samples were reacted with a goat secondary antibody (ZSGB‐BIO) at room temperature for 30 min. Signal detection was performed using DAB (ZSGB‐BIO), followed by hematoxylin staining. Slides were then dehydrated, coverslipped, and scanned using a NanoZoomer digital slide scanner (Hamamatsu). An immunoreactive score (IRS) was used to evaluate staining: IRS = intensity (negative 0, weakly positive = 1, positive = 2, strongly positive = 3) × proportion of positive cells (0%–5% = 0, 6%–25% = 1, 26%–50% = 2, 51%–75% = 3, 75%–100% = 4) for NNMT^+^, FN1^+^or pPI3K^+^ cells.

### 5.14 Statistical Analysis

Data are presented as the mean ± standard error of the mean (SEM), unless otherwise indicated. If the data were normally distributed and met the assumption of homogeneity, significance was assessed using Student's *t*‐test, multiple unpaired *t*‐tests, one‐way ANOVA followed by Dunnett's multiple comparisons test, or two‐way ANOVA followed by Sidák's multiple comparisons test. When the variable did not conform to a normal distribution, the Kruskal‐Wallis, Wilcoxon signed‐rank test, or the Mann–Whitney *U* test was used for data. Kaplan–Meier survival analyses were conducted using log‐rank tests. The experiments in cells were performed in triplicate using independent biological replicates. The statistical analyses were done with GraphPad Prism (version 9.5.1), and significance was defined as *p* < 0.05. (*p < 0.05; **p < 0.01; ***p < 0.001; ns, not significant.)

## Author Contributions

Lingling Wang drafted the manuscript, designed and performed the assays. Yueyang Wang reviewed the literature and collected patient samples. Qizheng Han analyzed the single‐cell data. Xiao Zhou conducted some of the animal experiments. Chenxia Wu contributed to the experimental methods. Honghe Zhang, Zhiyong Liang, and Maode Lai designed the study, provided experimental methods, and revised the manuscript. All authors have read and approved the final manuscript.

## Funding

This research was funded by the CAMS Innovation Fund for Medical Sciences (CIFMS, 2019‐I2M‐5‐044) and the National Key Clinical Specialty Construction Project (U114000).

## Ethics Statement

This study was approved by the Institutional Ethics Committee of the First Affiliated Hospital, Zhejiang University School of Medicine (No. 2021IIT923). The animal experiments were approved by the Laboratory Animal Welfare and Ethics Review Committee, Zhejiang University [ZJU20240223].

## Conflicts of Interest

The authors declare no conflicts of interest.

## Supporting information




**Supporting Figure 1**: NNMT localization and serum expression in ccRCC. (A) Flowchart of key molecule screening based on multi‐omics analysis. (B) Immunofluorescence staining showing the subcellular localization of NNMT. (C) ELISA analysis of serum NNMT expression levels in ccRCC patients and healthy controls. (D) Baseline characteristics and serum NNMT expression levels in healthy controls and ccRCC patients. Data are presented as mean ± standard deviation (SD). Significance was assessed using the Wilcoxon test (C, D). (ns, not significant). TCGA, The Cancer Genome Atlas; CPTAC, Clinical Proteomic Tumor Analysis Consortium; ccRCC, clear cell renal cell carcinoma; DEG, differentially expressed gene; DEP, differentially expressed protein; FDR, false discovery rate; HR, hazard ratio; Cox, Cox proportional hazards regression analysis; OS, overall survival; AUC, area under the curve.
**Supporting Figure 2**: Phenotypic changes in ccRCC cells following NNMT siRNA knockdown. (A–D) Validation of gene knockdown efficiency in 786‐O and ACHN cells at the mRNA and protein levels following siRNA transfection. (E, F) Migration and invasion assays of 786‐O (E) and ACHN (F) cells following siRNA transfection. (G, H) Quantification of the area migrated and invaded cells on the lower surface of Transwell chambers in 786‐O (G) and ACHN (H) cells. Significance was assessed using one‐way ANOVA followed by Dunnett's multiple comparisons test. (***p < 0.001). ccRCC, Clear cell renal cell carcinoma.
**Supporting Figure 3**: Effects of NAM or MNAM treatments on cellular functions. (A) IC_50_ of NAM in 786‐O cells. (B, C) Levels of total NAD and NAD^+^ in 786‐O cells after 48 h of NAM supplement. (D) mRNA expression in 786‐O cells after NAM supplementation. (E) NNMT protein expression in ccRCC cells following NAM supplement at high concentrations. (F, G) Levels of total NAD, NAD^+^ in 786‐O cells after NNMT knockdown. (H, I) Cell migration and invasion assays were performed in ccRCC cells after NAM treatment for 48 h at high concentrations. (J) IC_50_ of MNAM in 769‐P and OS‐RC‐2 cells. (K, L) Cell migration and invasion assays in ccRCC cells after MNAM treatment for 48 h at high concentrations. Significance was assessed using one‐way ANOVA followed by Dunnett's multiple comparisons test. (*p < 0.05; ***p < 0.001; ns, not significant).
**Supporting Figure 4**: NNMT promotes FN1 expression via H3K9me3. (A) Kaplan–Meier survival curves of ECM pathway‐related genes. (B) H3K9me3 protein levels in OS‐RC‐2 cells following NNMT knockout (NNMT‐KO). (C) ChIP–qPCR analysis of H3K9me3 enrichment at the FN1 promoter following NNMT‐KO in OS‐RC‐2 cells. (D) H3K9me3 modification and FN1 expression after supplementation with SAMe. (E) Schematic of two primer sites in the COL1A1 promoter region. (F) ChIP–qPCR analysis of H3K9me3 enrichment at the COL1A1 promoter in OS‐RC‐2 cells. (G) Co‐IP assay in HEK293T cells to assess whether exogenously overexpressed NNMT directly interacts with key m6A regulatory proteins. (H, I) RT‐qPCR analysis of H3K9 methyltransferase expression in different cell lines. (J) GSEA analysis of DEGs highlighting significantly enriched canonical pathways. (K) EMT‐related marker changes in 786‐O and OS‐RC‐2 cells. (L) Western blotting of FN1 downstream signaling proteins pFAK and pPI3K. Kaplan–Meier survival analyses were conducted with log‐rank tests (A), Student's *t*‐test was used for analysis (C, F), and multiple unpaired *t*‐tests followed by two‐stage step‐up FDR correction (Benjamini, Krieger, and Yekutieli) were used for (H) and (I). (**p < 0.01; ***p < 0.001; ns, not significant). ChIP, chromatin immunoprecipitation; SAMe, S‐adenosyl‐L‐methionine disulfate tosylate; Co‐IP, co‐immunoprecipitation; GSEA, Gene Set Enrichment Analysis; DEGs, differentially expressed genes.
**Supporting Figure 5**: FN1 rescue experiments in ccRCC cells. (A, B) Knockdown efficiency of FN1 in 786‐O and OS‐RC‐2 Mock and NNMT‐KO cells. (C, D) Transwell migration and invasion assays following FN1 knockdown in Mock and NNMT‐KO cells. (E, F) Transwell migration and invasion assays after exogenous supplementation with recombinant FN1 protein. Student's t‐test was used for analysis in (A–D), and one‐way ANOVA followed by Dunnett's multiple comparisons test was used in (E, F). (*p < 0.05; ***p < 0.001; ns, not significant).
**Supporting Figure 6**: NNMT upregulation links FN1‐associated signaling in ccRCC. (A) Correlation analysis of FN1 and NNMT expression in the GSE46699 and GSE53757 datasets. (B) NNMT mRNA expression in tumor and normal samples from the GEO datasets. (C) Representative IHC staining of NNMT in ccRCC at different stages from our cohort. (D, E) IHC staining of pPI3K in tumor tissues from transplanted OS‐RC‐2 Mock and NNMT‐KO tumor‐bearing mice (D) and from NNMTi‐treated mice(E). (F, G) Quantification of pPI3K staining corresponding to panels (D) and (E). Significance was assessed using the Wilcoxon test (B), Student's *t*‐test (F), or one‐way ANOVA followed by Dunnett's multiple comparisons test (G). (**p < 0.01; ***p < 0.001; ns, not significant). IHC, Immunohistochemical; NNMTi, NNMT inhibitor (HY‐131042, MCE).
**Supporting Table 1**: Comparison of clinicopathological characteristics in ccRCC patients (*n* = 74).
**Supporting Data 1**: Patient baseline characteristics for serum NNMT levels measured by ELISA (Figure S1C, D).
**Supporting Data 2**: Patient baseline characteristics for NNMT and FN1 expression (Figure 6A–D).
**Supporting Data 3**: Patient baseline characteristics for NNMT immunohistochemistry in human tissues (Figure 6F, G).

## Data Availability

The transcriptomic data for patients with ccRCC were obtained from TCGA through the GDC Data Portal (data accessed in March 2024). The transcriptomic survival data were retrieved from UCSC Xena. Proteomic expression matrices for ccRCC were retrieved from CPTAC (data accessed in March 2024), and clinical data for the patients were acquired from the original CPTAC publication reporting this cohort [47]. RNA sequencing datasets were obtained from the GEO database (GSE171306 for single‐cell RNA, GSE46699 and GSE53757 for mRNA sequencing). The RNA‐seq and ChIP‐Seq data of OS‐RC‐2 cells were uploaded to the Sequence Read Archive (SRA) database (PRJNA1329281, PRJNA1426512).
