# Supplementary material for: Nicotinamide N‐Methyltransferase Epigenetically Activates Fibronectin 1 Through H3K9me3 Remodeling in Clear Cell Renal Cell Carcinoma
Source: MedComm (2020). 2026 Jul 5;7(7):e70831. doi: 10.1002/mco2.70831 (PMC13334132; doi:10.1002/mco2.70831)
Supplement: Supplementary file 1 — Supporting Figure 1: NNMT localization and serum expression in ccRCC. (A) Flowchart of key molecule screening based on multi‐omics analysis. (B) Immunofluorescence staining showing the subcellular localization of NNMT. (C) ELISA analysis of serum NNMT expression levels in ccRCC patients and healthy controls. (D) Baseline characteristics and serum NNMT expression levels in healthy controls and ccRCC patients. Data are presented as mean ± standard deviation (SD). Significance was assessed using the Wilcoxon test (C, D). (ns, not significant). TCGA, The Cancer Genome Atlas; CPTAC, Clinical Proteomic Tumor Analysis Consortium; ccRCC, clear cell renal cell carcinoma; DEG, differentially expressed gene; DEP, differentially expressed protein; FDR, false discovery rate; HR, hazard ratio; Cox, Cox proportional hazards regression analysis; OS, overall survival; AUC, area under the curve. Supporting Figure 2: Phenotypic changes in ccRCC cells following NNMT siRNA knockdown. (A–D) Validation of gene knockdown efficiency in 786‐O and ACHN cells at the mRNA and protein levels following siRNA transfection. (E, F) Migration and invasion assays of 786‐O (E) and ACHN (F) cells following siRNA transfection. (G, H) Quantification of the area migrated and invaded cells on the lower surface of Transwell chambers in 786‐O (G) and ACHN (H) cells. Significance was assessed using one‐way ANOVA followed by Dunnett's multiple comparisons test. (***p < 0.001). ccRCC, Clear cell renal cell carcinoma. Supporting Figure 3: Effects of NAM or MNAM treatments on cellular functions. (A) IC50 of NAM in 786‐O cells. (B, C) Levels of total NAD and NAD+ in 786‐O cells after 48 h of NAM supplement. (D) mRNA expression in 786‐O cells after NAM supplementation. (E) NNMT protein expression in ccRCC cells following NAM supplement at high concentrations. (F, G) Levels of total NAD, NAD+ in 786‐O cells after NNMT knockdown. (H, I) Cell migration and invasion assays were performed in ccRCC cells after NAM treatme [file MCO2-7-e70831-s001.docx]

**Supplementary Materials**

**Nicotinamide N-methyltransferase Epigenetically Activates Fibronectin 1 through H3K9me3 Remodeling in Clear Cell Renal Cell Carcinoma**

Lingling Wang1,2,3**#**, Yueyang Wang1,2,3**#**, Qizheng Han3, Xiao Zhou3, Chenxia Wu3, Honghe Zhang2,3, Zhiyong Liang1*****, Maode Lai2,3*****

1 Department of Pathology, Peking Union Medical College Hospital, Chinese Academy of Medical Sciences and Peking Union Medical College, Beijing, China

2 Research Unit of Intelligence Classification of Tumor Pathology and Precision Therapy, Chinese Academy of Medical Sciences (2019RU042) and Zhejiang University School of Medicine, Hangzhou, China.

3 Department of Pathology, Zhejiang University School of Medicine, Hangzhou, China.

**Correspondence:**

Zhiyong Liang (liangzy@pumch.cn) | Maode Lai (lmd@zju.edu.cn)

**Running title:** NNMT regulates FN1 through H3K9me3 in ccRCC

**This file includes:**

**Supplementary Methods**

**Supplementary Figures 1-6**

**Supplementary Table 1**

**Oligonucleotide sequences**

**Demographic data**

**Supplementary Methods**

**Immunofluorescence Assay**

Cells were cultured on high-optical-clear-bottom dishes. They were fixed for 20 minutes and then treated with 0.1% Triton X-100 for an additional 20 minutes. Following blocked for 1 hour with 10% FBS, cells were added primary antibodies and stored at 4°C. Cells were covered with Alexa Fluor 647 (Invitrogen, A32728) or Alexa Fluor 546 (Invitrogen, A-11010) secondary antibodies for 1 hour in the dark, followed by staining with DAPI (Invitrogen, D1306) for 20 minutes. Representative images were analyzed using FV3000 (Olympus).

**ELISA Assay**
Serum Nicotinamide N-methyltransferase (NNMT) protein levels of human and S-Adenosyl Methionine (SAM) of cell lysis were measured using ELISA kits (Cloud-Clone, SEH530Hu for NNMT and Cat #CEG414Ge for SAM). Kits were equilibrated to room temperature prior to use. All procedures, including sample incubation, washing, and color development with substrate, were performed. A microplate detector was used to quantify protein expression (450 nm).

**Co-Immunoprecipitation (Co-IP)**

HEK293T were transfected with FLAG-NNMT overexpression plasmid. After, cells were shaved off, and protein complexes were immunoprecipitated using anti-HA (Yoche, AYD02-100) or anti-FLAG (Sigma-Aldrich, F1804) antibodies. IP were analyzed by WB for m6A regulatory proteins to assess direct interactions.

**ChIP-Seq (Chromatin Immunoprecipitation sequencing)**

Library construction and sequencing were performed by Sangon Biotech. DNA libraries were prepared using a YEASEN DNA Library Preparation Kit (Shanghai, China) following the standard protocol, including end repair, 3′-adenylation, adaptor ligation, and PCR indexing. Library concentration and quality were evaluated by Qubit fluorometry and 2% agarose gel electrophoresis. Pooled libraries were sequenced on a DNBSEQ-T7 platform (BGI, Shenzhen, China) using a 2 × 150 bp paired-end configuration. Raw reads were processed with Trimmomatic (v0.39) to remove adaptor sequences and low-quality bases, followed by alignment to the reference genome using BWA (v0.7.17). Peak calling was performed with MACS2 (v2.1.1), using the corresponding input samples for background normalization.

**Supplementary Figures**

**
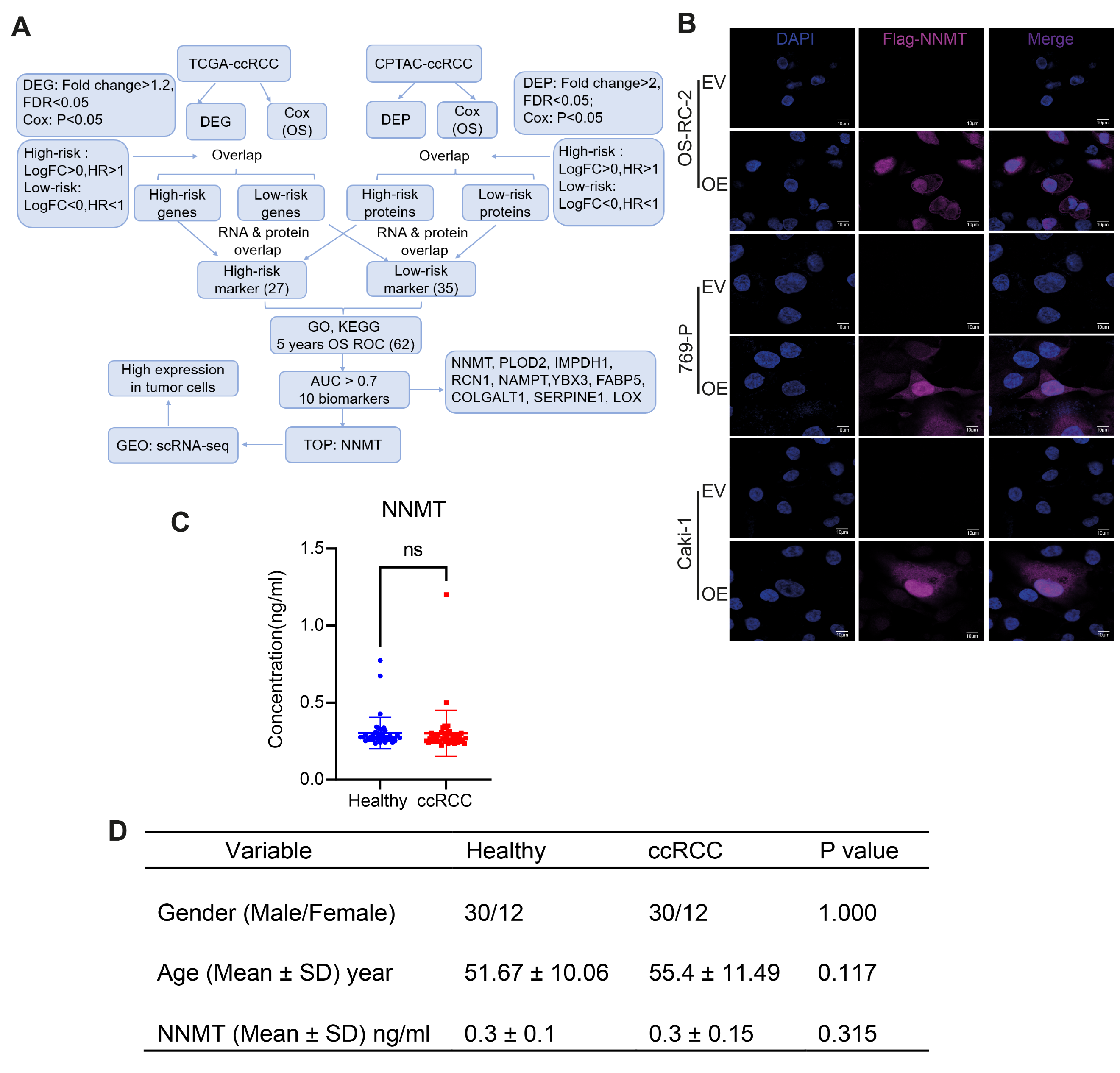
**

**FIGURE** **S1 |** NNMT localization and serum expression in ccRCC. (A) Flowchart of key molecule screening based on multi-omics analysis. (B) Immunofluorescence staining showing the subcellular localization of NNMT. (C) ELISA analysis of serum NNMT expression levels in ccRCC patients and healthy controls. (D) Baseline characteristics and serum NNMT expression levels in healthy controls and ccRCC patients. Data are presented as mean ± standard deviation (SD). Significance was assessed using Wilcoxon test (C, D). (ns, not significant). TCGA, The Cancer Genome Atlas; CPTAC, Clinical Proteomic Tumor Analysis Consortium; ccRCC, Clear cell renal cell carcinoma; DEG, Differentially expressed gene; DEP, Differentially expressed protein; FDR, False discovery rate; HR, Hazard ratio; Cox, Cox proportional hazards regression analysis; OS, Overall survival; AUC, Area under the curve.

**
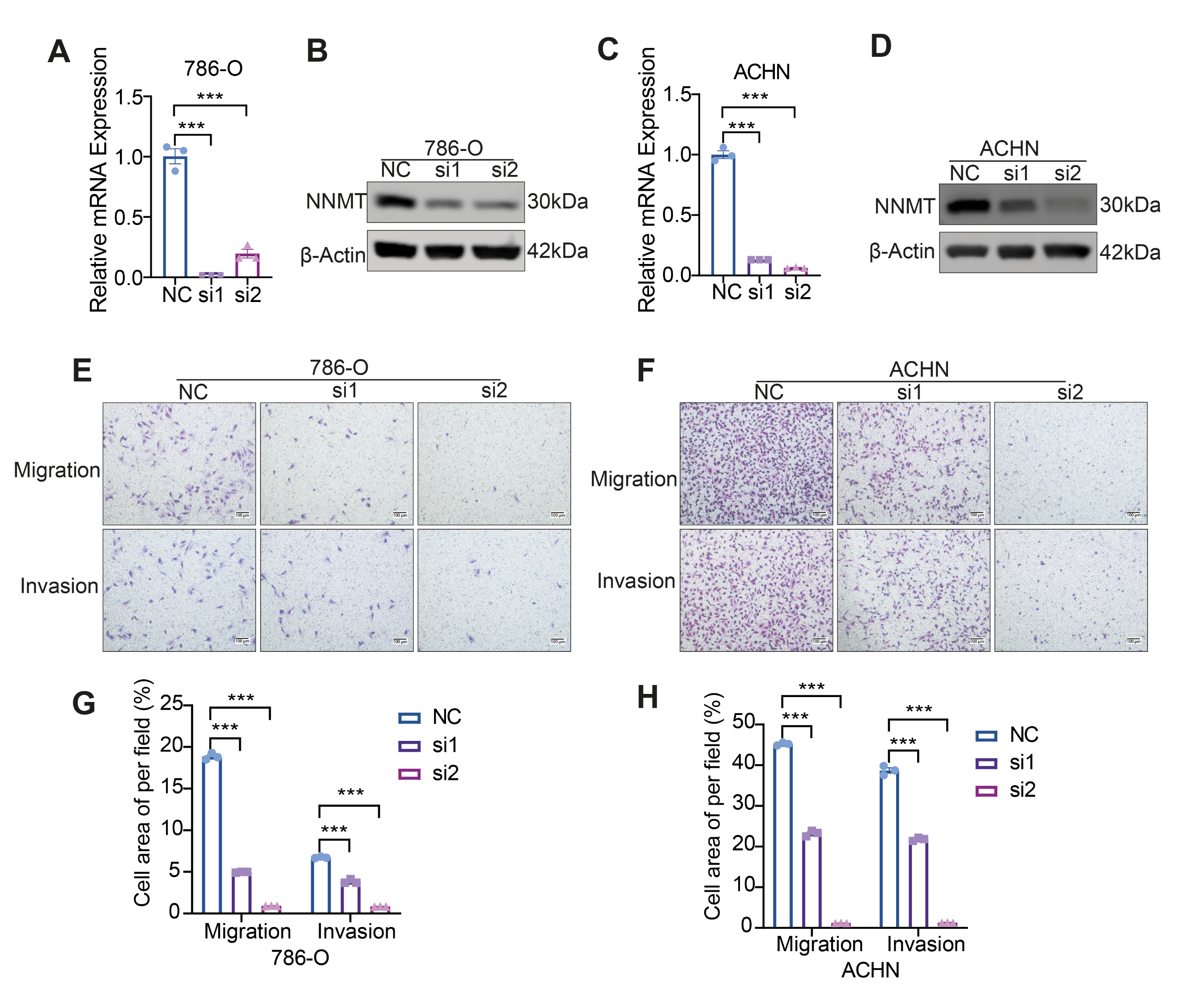
FIGURE S2 |** Phenotypic changes in ccRCC cells following NNMT siRNA knockdown. (A-D) Validation of gene knockdown efficiency in 786-O and ACHN cells at the mRNA and protein levels following siRNA transfection. (E, F) Migration and invasion assays of 786-O (E) and ACHN (F) cells following siRNA transfection. (G, H) Quantification of the area migrated and invaded cells on the lower surface of Transwell chambers in 786-O (G) and ACHN (H) cells. Significance was assessed using one-way ANOVA followed by Dunnett's multiple comparisons test. (****P*<0.001). ccRCC, Clear cell renal cell carcinoma.

**
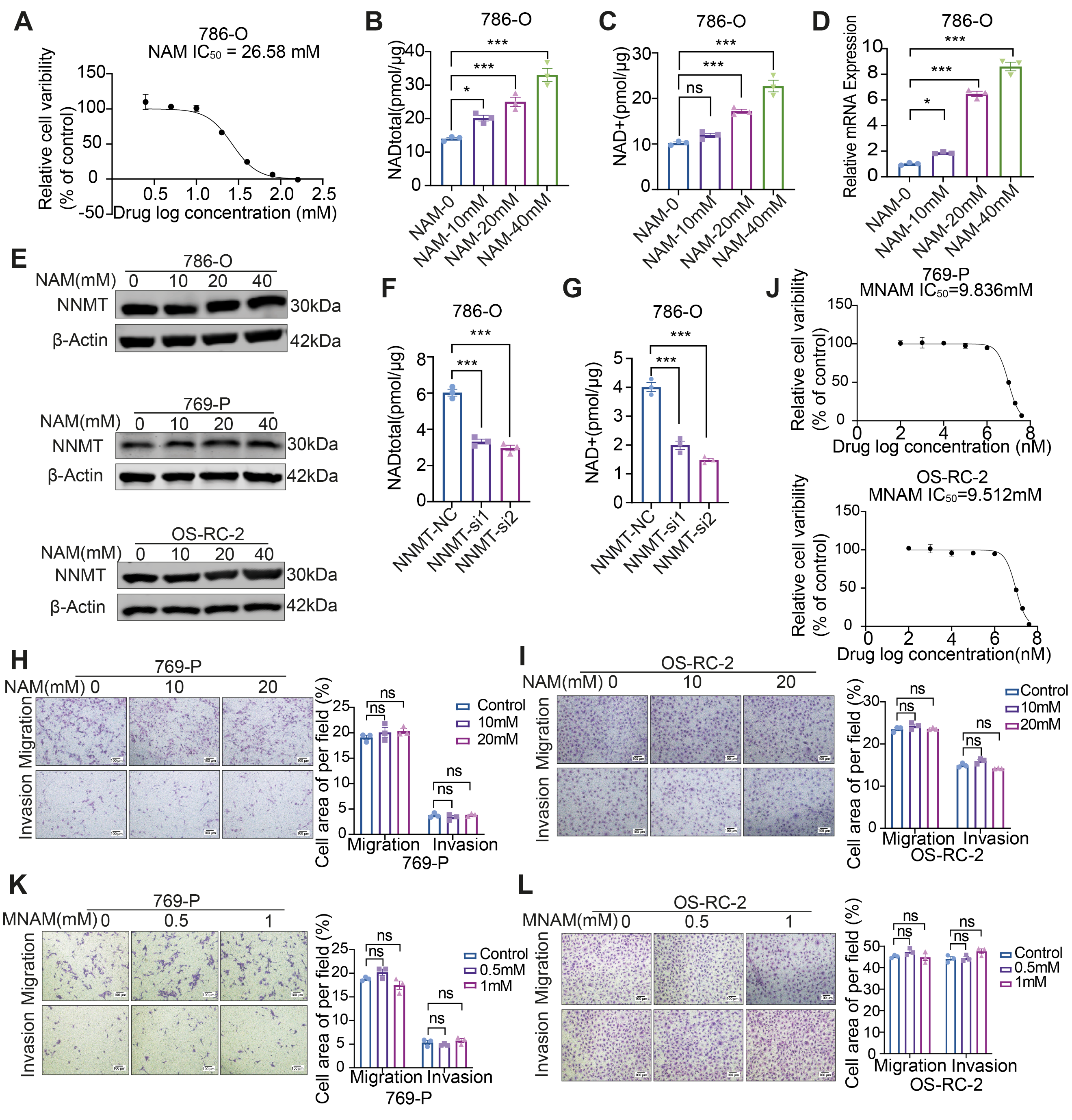
FIGURE S3 |** Effects of NAM or MNAM treatments on cellular functions. (A) IC_50_ of NAM in 786-O cell. (B, C) Levels of total NAD and NAD⁺ in 786-O cell after 48 hours of NAM supplement. (D) mRNA expression in 786-O cell after NAM supplementation. (E) NNMT protein expression in ccRCC cells following NAM supplement at high concentrations. (F, G) Levels of total NAD, NAD⁺ in 786-O cells after NNMT knockdown. (H, I) Cell migration and invasion assays were performed in ccRCC cells after NAM treatment for 48h in high concentration. (J) IC_50_ of MNAM in 769-P and OS-RC-2 cells. (K, L) Cell migration and invasion assays in ccRCC cells after MNAM treatment for 48h at high concentrations. Significance was assessed using one-way ANOVA followed by Dunnett's multiple comparisons test. (**P*<0.05; ****P*<0.001; ns, not significant).

**
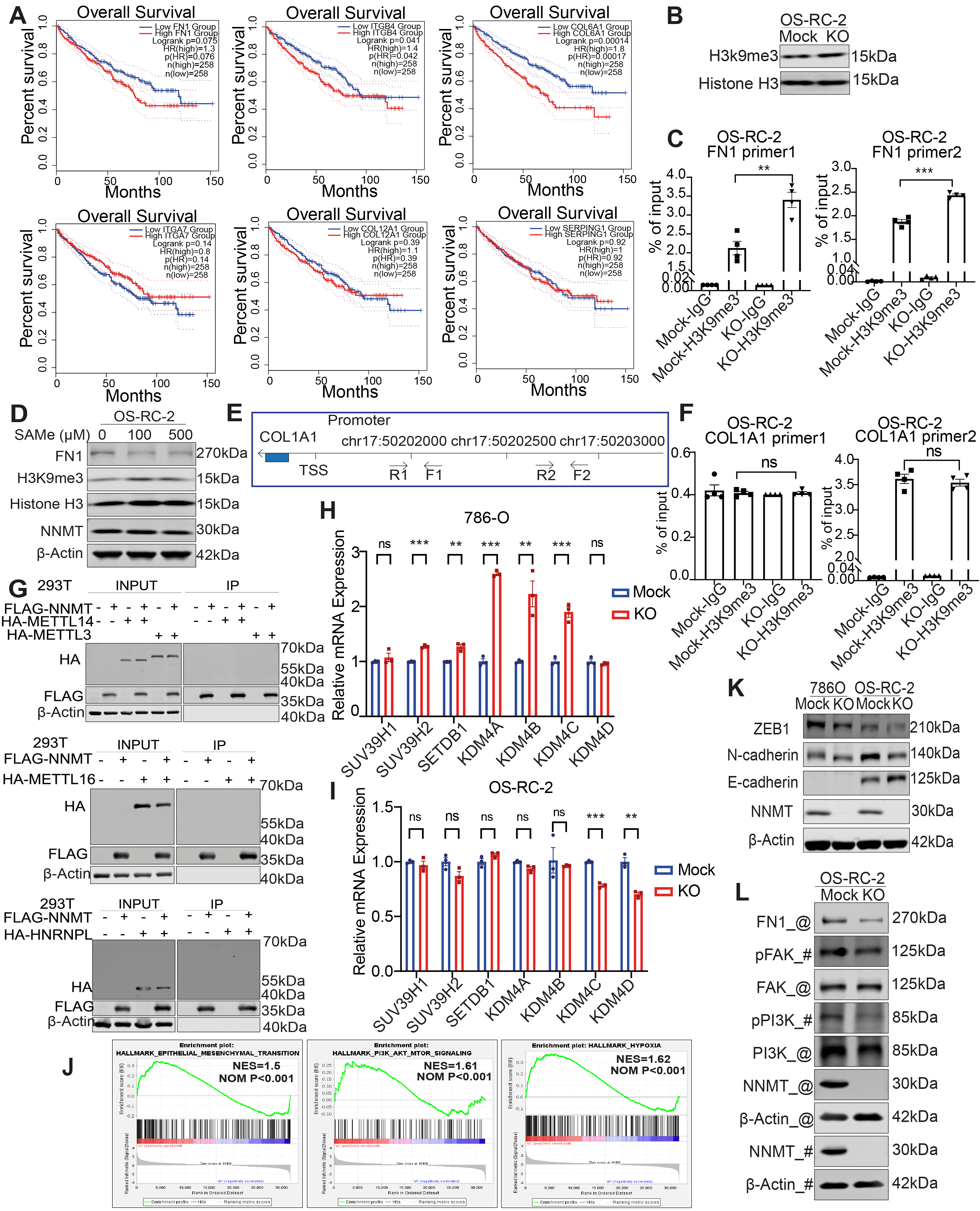
**

**FIGURE S4 |** NNMT promotes FN1 expression via H3K9me3. (A) Kaplan–Meier survival curves of ECM pathway-related genes. (B) H3K9me3 protein levels in OS-RC-2 cells following NNMT knockout (NNMT-KO). (C) ChIP–qPCR analysis of H3K9me3 enrichment at the FN1 promoter following NNMT-KO in OS-RC-2 cells. (D) H3K9me3 modification and FN1 expression after supplementation with SAMe. (E) Schematic of two primer sites in the COL1A1 promoter region. (F) ChIP–qPCR analysis of H3K9me3 enrichment at the COL1A1 promoter in OS-RC-2 cells. (G) Co-IP assay in HEK293T cells to assess whether exogenously overexpressed NNMT directly interacts with key m6A regulatory proteins. (H, I) RT-qPCR analysis of H3K9 methyltransferase expression in different cell lines. (J) GSEA analysis of DEGs highlighting significantly enriched canonical pathways. (K) EMT-related marker changes in 786-O and OS-RC-2 cells. (L) Western blotting of FN1 downstream signaling proteins pFAK and pPI3K. Kaplan–Meier survival analyses were conducted with log-rank tests (A), Student’s *t*-test was used for analysis (C, F), and multiple unpaired *t*-tests followed by two-stage step-up FDR correction (Benjamini, Krieger, and Yekutieli) were used for H and I. (***P*<0.01; ****P*<0.001; ns, not significant). ChIP, Chromatin Immunoprecipitation; SAMe, S-Adenosyl-L-methionine disulfate tosylate, Co-IP, Co-immunoprecipitation; GSEA, Gene set enrichment analysis; DEGs, Differentially expressed genes.

**
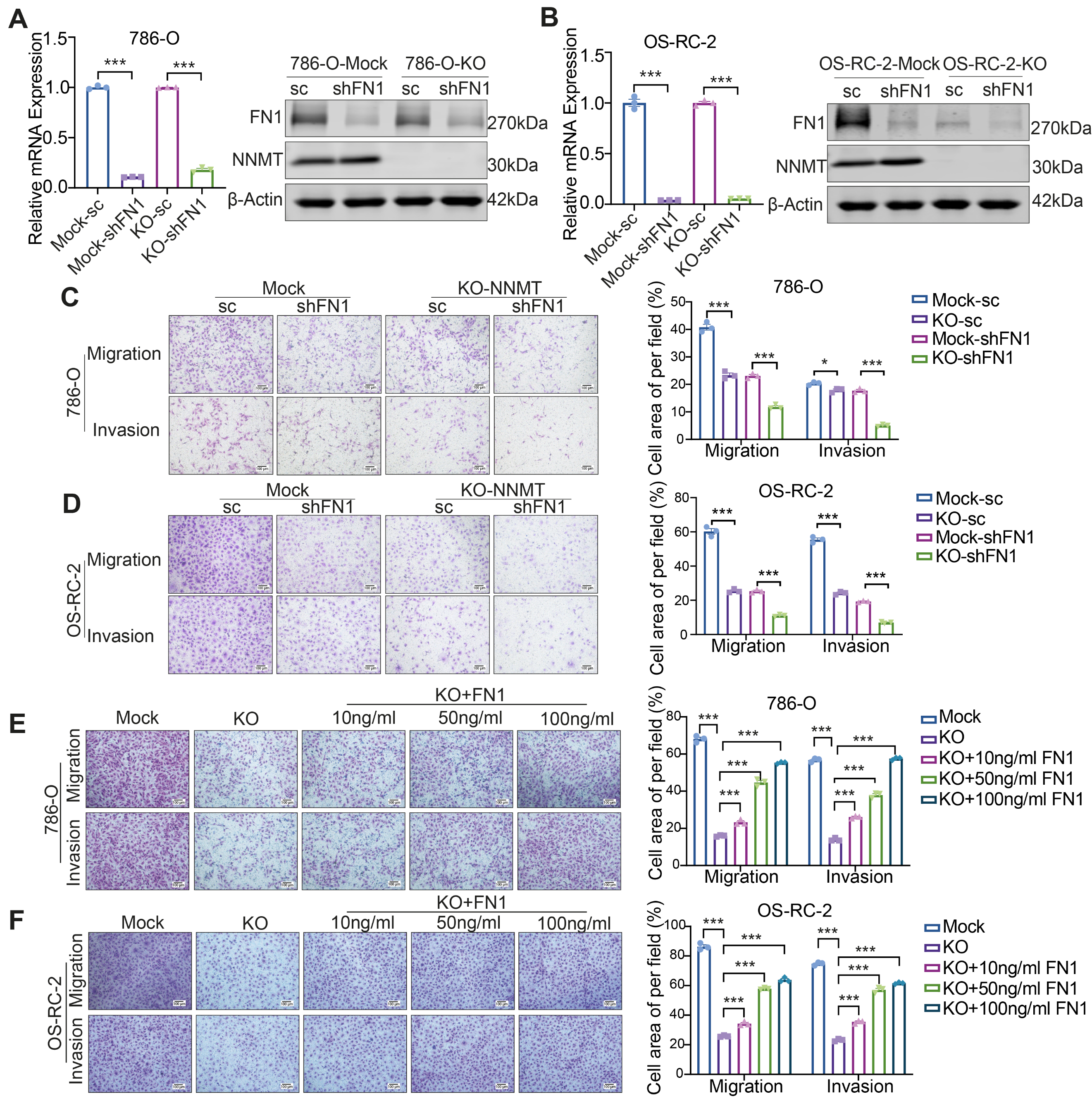
**

**FIGURE S5 |** FN1 rescue experiments in ccRCC cells. (A, B) Knockdown efficiency of FN1 in 786-O and OS-RC-2 Mock and NNMT-KO cells. (C, D) Transwell migration and invasion assays following FN1 knockdown in Mock and NNMT-KO cells. (E, F) Transwell migration and invasion assays after exogenous supplementation with recombinant FN1 protein. Student’s t-test was used for analysis in (A–D), and one-way ANOVA followed by Dunnett’s multiple comparisons test was used in (E, F). (* *P*<0.05; ****P*<0.001; ns, not significant).

**
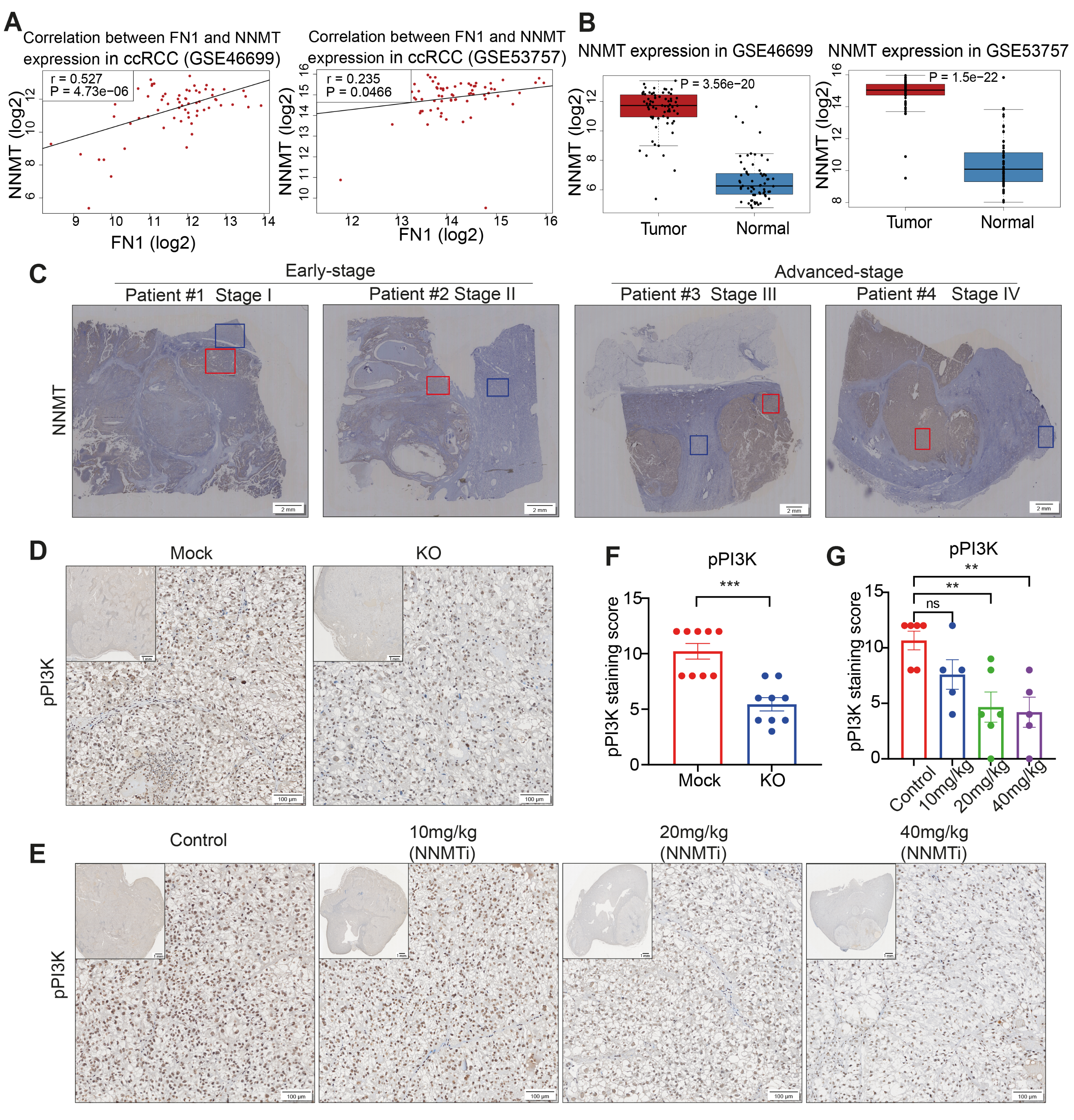
**

**FIGURE S6 |** NNMT upregulation links FN1-associated signaling in ccRCC. (A) Correlation analysis of FN1 and NNMT expression in the GSE46699 and GSE53757 datasets. (B) NNMT mRNA expression in tumor and normal samples from GEO datasets. (C) Representative IHC staining of NNMT in ccRCC at different stages from our cohort. (D, E) IHC staining of pPI3K in tumor tissues from transplanted OS-RC-2 Mock and NNMT-KO tumor-bearing mice (D) and from NNMTi-treated mice(E). (F, G) Quantification of pPI3K staining corresponding to panels (D) and (E). Significance was assessed using Wilcoxon test (B), Student’s *t*-test (F), or one-way ANOVA followed by Dunnett's multiple comparisons test (G). (***P*<0.01; ****P*<0.001; ns, not significant). IHC, Immunohistochemical; NNMTi, NNMT inhibitor (HY-131042, MCE).

**Supplementary Tables**

**TABLE S1.** Comparison of clinicopathological characteristics in ccRCC patients (n=74).

| **Variable** | **NNMT Low** | **NNMT High** | **P value** |
| --- | --- | --- | --- |
| Age (mean ± SD) (year) | 54.8 ± 14.2 | 57.6 ± 12.9 | 0.403 |
| Gender (n, %) | Female (n=10, 29.4%) Male (n=24, 70.6%) | Female (n=13, 32.5%) Male (n=27, 67.5%) | 0.973 |
| TNM Stage (n, %) | Early-stage (n= 30, 88.2%) Advanced-stage (n=4, 11.8%) | Early-stage (n=8, 20%) Advanced-stage (n=32, 80%) | <0.001 |
| Patients were classified into NNMT high and low expression groups according to the mean Immunoreactive Score (IRS). Age was analyzed by the Wilcoxon rank-sum test, Gender by the Chi-square test, and TNM Stage by Fisher’s exact test. | | | |

**Oligonucleotide Sequences in this study.**

| **qPCR** | | | |
| --- | --- | --- | --- |
| **Primer** | **Species** | **Forward sequence (5’-3’)** | **Reverse sequence (5’-3’)** |
| **COL6A1** | **Human** | **ACAGTGACGAGGTGGAGATCA** | **GATAGCGCAGTCGGTGTAGG** |
| **COL6A2** | **Human** | **GACTCCACCGAGATCGACCA** | **CTTGTAGCACTCTCCGTAGGC** |
| **HSPG2** | **Human** | **GTGTGGTGTTCATCAAGGAGC** | **GGGAGAGGTGACGTAGGAGG** |
| **THBS2** | **Human** | **GACACGCTGGATCTCACCTAC** | **GAAGCTGTCTATGAGGTCGCA** |
| **LAMA1** | **Human** | **GTGATGGCAACAGCGCAAA** | **GACCCAGTGATATTCTCTCCCA** |
| **LAMA5** | **Human** | **CCCACCGAGGACCTTTACTG** | **GGTGTGCCTTGTTGCTGTT** |
| **ITGA7** | **Human** | **CTGACTCCATGTTCGGGATCA** | **CACCTGTGAAGGTTTGGCG** |
| **ITGB4** | **Human** | **CTCCACCGAGTCAGCCTTC** | **CGGGTAGTCCTGTGTCCTGTA** |
| **SERPINF2** | **Human** | **GTGCCCGTGGAAATGATGC** | **AAAGTGGGTGGGTACAAGGAC** |
| **SERPING1** | **Human** | **GGGATGCTTTGGTAGATTTCTCC** | **GAGGATGCTCTCCAGGTTTGT** |
| **COL12A1** | **Human** | **AGCTGAGGCAGACATTGTGTT** | **CCTCCTTTGTACGGCAAGTTT** |
| **FN1** | **Human** | **GAGAATAAGCTGTACCATCGCAA** | **CGACCACATAGGAAGTCCCAG** |
| **NNMT** | **Human** | **AGCTGGAGAAGTGGCTGAAG** | **TGGACCCTTGACTCTGTTCC** |
| **β-actin** | **Human** | **CATGTACGTTGCTATCCAGGC** | **CTCCTTAATGTCACGCACGAT** |
| **SUV39H1** | **Human** | **CGTTAGCCGTGGGGAAAGAT** | **CGCACAGGTACTCGACTTCA** |
| **SUV39H2** | **Human** | **TGAGGTGGAATACTTGTGTGACT** | **AAGCAGTAACGGGCACTTCA** |
| **SETDB1** | **Human** | **GTGAGTCTGGGGTCTGGTTG** | **TGCTGCATCCAAACCAATGC** |
| **KDM4A** | **Human** | **GGAGCTGATGCCACCTCTTT** | **ATACTTTGCCACAGGGGCTC** |
| **KDM4B** | **Human** | **CACGCCGCCTCAGGAAC** | **CACTTCTGGATGGCGAGGTT** |
| **KDM4C** | **Human** | **CGAGGTGGAAAGTCCTCTGAA** | **GGGCTCCTTTAGACTCCATGTAT** |
| **KDM4D** | **Human** | **CAAGAGCTGAGCACCCAGAA** | **GTGGGAGTGAAGAGCACACA** |
| **KOvalidateNNMT** | **Human** | **CACTGCCATGAGATGCCTGA** | **GCTCTCGCTCTCTCGTTACC** |
| **siRNA/shRNA/sgRNA** | | | |
| **Primer** | **Species** | **Sequence** |  |
| **si1-hNNMT** | **Human** | **CCUCUCUGCUUGUGAAUCCUUTT** |  |
| **si2-hNNMT** | **Human** | **GCUCAAGAGCAGCUACUACAUTT** |  |
| **sh1-hNNMT** | **Human** | **CCTCTCTGCTTGTGAATCCTT** |  |
| **sh3-hNNMT** | **Human** | **ACCCTCGGGATTACCTAGAAA** |  |
| **sh-hFN** | **Human** | **ACACTATCAGATAAATCAA** |  |
| **SC** | **Human** | **ATCTCGCTTGGGCGAGAGTAAG** |  |
| **NC** | **Human** | **UUCUCCGAACGUGUCACGUTT** |  |
| **NNMT-sgRNA** | **Human** | **CACCGAGTGACGACGATCTCCTTAA** |  |
| **ChIP-qPCR** | | | |
| **Primer** | **Species** | **Forward sequence (5’-3’)** | **Reverse sequence (5’-3’)** |
| **FN1-primer1** | **Human** | **CTTAACAGCTGCAAGGTCGTG** | **GCTGGACTTGTGTGAAGCGA** |
| **FN1-primer2** | **Human** | **ACCCACCCGGCTTTTAATGA** | **TTCGTGTCCTTTCCCACGTT** |
| **COL1A1-primer1** | **Human** | **GGTCCTCAGCATGCCTCTTT** | **GGGGAGACTTCCAAAGGTGG** |
| **COL1A1-primer2** | **Human** | **GGTACAACTGGAGAAGGGGC** | **TAGAGACCCTCCTTCCCAGC** |

**Demographic data files**

**Data S1**

Patient baseline characteristics for serum NNMT levels measured by ELISA (Figure S1C-D).

| **group (Normal/Tumor)** | **rank** | **ID** | **gender** | **age (year)** |
| --- | --- | --- | --- | --- |
| **N** | **N1** | **F197** | **female** | **58** |
| **N** | **N2** | **F200** | **female** | **63** |
| **N** | **N3** | **F202** | **female** | **63** |
| **N** | **N4** | **F205** | **female** | **65** |
| **N** | **N5** | **F208** | **female** | **64** |
| **N** | **N6** | **F214** | **female** | **64** |
| **N** | **N7** | **M047** | **male** | **34** |
| **N** | **N8** | **M051** | **male** | **38** |
| **N** | **N9** | **M057** | **male** | **33** |
| **N** | **N10** | **M098** | **male** | **49** |
| **N** | **N11** | **M106** | **male** | **50** |
| **N** | **N12** | **M117** | **male** | **44** |
| **N** | **N13** | **M121** | **male** | **47** |
| **N** | **N14** | **M125** | **male** | **48** |
| **N** | **N15** | **M126** | **male** | **42** |
| **N** | **N16** | **M221** | **male** | **54** |
| **N** | **N17** | **M235** | **male** | **52** |
| **N** | **N18** | **M244** | **male** | **60** |
| **N** | **N19** | **M262** | **male** | **57** |
| **N** | **N20** | **M274** | **male** | **58** |
| **N** | **N21** | **M287** | **male** | **55** |
| **N** | **N22** | **M306** | **male** | **66** |
| **N** | **N23** | **M318** | **male** | **66** |
| **N** | **N24** | **M344** | **male** | **62** |
| **N** | **N25** | **M347** | **male** | **61** |
| **N** | **N26** | **M363** | **male** | **63** |
| **N** | **N27** | **M388** | **male** | **63** |
| **N** | **N28** | **M389** | **male** | **63** |
| **N** | **N29** | **F059** | **female** | **49** |
| **N** | **N30** | **F063** | **female** | **48** |
| **N** | **N31** | **F067** | **female** | **49** |
| **N** | **N32** | **F068** | **female** | **48** |
| **N** | **N33** | **F070** | **female** | **50** |
| **N** | **N34** | **F071** | **female** | **49** |
| **N** | **N35** | **M013** | **male** | **29** |
| **N** | **N36** | **M023** | **male** | **38** |
| **N** | **N37** | **M026** | **male** | **35** |
| **N** | **N38** | **M060** | **male** | **47** |
| **N** | **N39** | **M066** | **male** | **43** |
| **N** | **N40** | **M080** | **male** | **48** |
| **N** | **N41** | **M082** | **male** | **46** |
| **N** | **N42** | **M083** | **male** | **49** |
| **T** | **T1** | **R-200** | **male** | **66** |
| **T** | **T2** | **R-207** | **male** | **35** |
| **T** | **T3** | **R-214** | **male** | **47** |
| **T** | **T4** | **R-217** | **male** | **49** |
| **T** | **T5** | **R-239** | **male** | **52** |
| **T** | **T6** | **R-246** | **male** | **54** |
| **T** | **T7** | **R-295** | **male** | **57** |
| **T** | **T8** | **R-297** | **female** | **53** |
| **T** | **T9** | **R-325** | **male** | **63** |
| **T** | **T10** | **R-326** | **male** | **60** |
| **T** | **T11** | **R-360** | **female** | **68** |
| **T** | **T12** | **R-387** | **female** | **58** |
| **T** | **T13** | **R-388** | **male** | **55** |
| **T** | **T14** | **R-394** | **male** | **44** |
| **T** | **T15** | **R-416** | **male** | **63** |
| **T** | **T16** | **R-432** | **male** | **63** |
| **T** | **T17** | **R-441** | **male** | **50** |
| **T** | **T18** | **R-452** | **male** | **38** |
| **T** | **T19** | **R-475** | **female** | **44** |
| **T** | **T20** | **R-482** | **female** | **81** |
| **T** | **T21** | **R-490** | **male** | **61** |
| **T** | **T22** | **R-560** | **male** | **58** |
| **T** | **T23** | **R-565** | **male** | **48** |
| **T** | **T24** | **R-575** | **female** | **58** |
| **T** | **T25** | **R-585** | **male** | **66** |
| **T** | **T26** | **R-588** | **male** | **42** |
| **T** | **T27** | **R-651** | **male** | **62** |
| **T** | **T28** | **R-710** | **male** | **35** |
| **T** | **T29** | **R-299** | **male** | **29** |
| **T** | **T30** | **R-300** | **male** | **68** |
| **T** | **T31** | **R-301** | **male** | **69** |
| **T** | **T32** | **R-317** | **female** | **67** |
| **T** | **T33** | **R-318** | **female** | **56** |
| **T** | **T34** | **R-330** | **female** | **79** |
| **T** | **T35** | **R-331** | **male** | **55** |
| **T** | **T36** | **R-332** | **male** | **69** |
| **T** | **T37** | **R-335** | **male** | **49** |
| **T** | **T38** | **R-337** | **female** | **62** |
| **T** | **T39** | **R-341** | **male** | **50** |
| **T** | **T40** | **R-345** | **male** | **48** |
| **T** | **T41** | **R-369** | **female** | **55** |
| **T** | **T42** | **R-370** | **female** | **41** |

Notes: ELISA, Enzyme-linked immunosorbent assay; N, Normal; T, Tumor.

**Data S2**

Patient baseline characteristics for NNMT and FN1 expression (Figure 6A-D).

| **ID** | **sex** | **age (year)** | **diagnosis** |
| --- | --- | --- | --- |
| **1** | **male** | **46** | **ccRCC** |
| **2** | **male** | **59** | **ccRCC** |
| **3** | **male** | **71** | **ccRCC** |
| **4** | **female** | **54** | **ccRCC** |
| **5** | **male** | **50** | **ccRCC** |
| **6** | **female** | **33** | **ccRCC** |
| **7** | **male** | **53** | **ccRCC** |
| **8** | **male** | **74** | **ccRCC** |
| **9** | **female** | **71** | **ccRCC** |
| **10** | **male** | **52** | **ccRCC** |
| **11** | **male** | **26** | **ccRCC** |
| **12** | **male** | **54** | **ccRCC** |
| **13** | **female** | **73** | **ccRCC** |
| **14** | **male** | **67** | **ccRCC** |
| **15** | **male** | **70** | **ccRCC** |
| **16** | **male** | **51** | **ccRCC** |
| **17** | **male** | **77** | **ccRCC** |
| **18** | **male** | **61** | **ccRCC** |
| **19** | **male** | **72** | **ccRCC** |
| **20** | **male** | **59** | **ccRCC** |
| **21** | **female** | **47** | **ccRCC** |
| **22** | **male** | **67** | **ccRCC** |

Notes: ccRCC, Clear Cell Renal Cell Carcinoma.

**Data S3**

Patient baseline characteristics for NNMT immunohistochemistry in human tissues (Figure 6F-G).

| **ID** | **age (year)** | **sex** | **stage_1** | **stage_2** | **histological_type** |
| --- | --- | --- | --- | --- | --- |
| **1** | **54** | **female** | **1** | **Early-stage** | **ccRCC** |
| **2** | **41** | **male** | **4** | **Advanced-stage** | **ccRCC** |
| **3** | **49** | **male** | **3** | **Advanced-stage** | **ccRCC** |
| **4** | **58** | **female** | **1** | **Early-stage** | **ccRCC** |
| **5** | **65** | **male** | **1** | **Early-stage** | **ccRCC** |
| **6** | **61** | **male** | **1** | **Early-stage** | **ccRCC** |
| **7** | **46** | **male** | **1** | **Early-stage** | **ccRCC** |
| **8** | **33** | **male** | **1** | **Early-stage** | **ccRCC** |
| **9** | **51** | **male** | **1** | **Early-stage** | **ccRCC** |
| **10** | **60** | **female** | **1** | **Early-stage** | **ccRCC** |
| **11** | **65** | **male** | **1** | **Early-stage** | **ccRCC** |
| **12** | **26** | **male** | **1** | **Early-stage** | **ccRCC** |
| **13** | **55** | **female** | **3** | **Advanced-stage** | **ccRCC** |
| **14** | **58** | **male** | **2** | **Early-stage** | **ccRCC** |
| **15** | **59** | **male** | **4** | **Advanced-stage** | **ccRCC** |
| **16** | **68** | **female** | **3** | **Advanced-stage** | **ccRCC** |
| **17** | **76** | **male** | **4** | **Advanced-stage** | **ccRCC** |
| **18** | **54** | **male** | **4** | **Advanced-stage** | **ccRCC** |
| **19** | **81** | **male** | **3** | **Advanced-stage** | **ccRCC** |
| **20** | **67** | **male** | **1** | **Early-stage** | **ccRCC** |
| **21** | **72** | **male** | **3** | **Advanced-stage** | **ccRCC** |
| **22** | **56** | **male** | **3** | **Advanced-stage** | **ccRCC** |
| **23** | **35** | **male** | **3** | **Advanced-stage** | **ccRCC** |
| **24** | **75** | **male** | **3** | **Advanced-stage** | **ccRCC** |
| **25** | **53** | **male** | **2** | **Early-stage** | **ccRCC** |
| **26** | **45** | **male** | **1** | **Early-stage** | **ccRCC** |
| **27** | **31** | **female** | **1** | **Early-stage** | **ccRCC** |
| **28** | **42** | **male** | **3** | **Advanced-stage** | **ccRCC** |
| **29** | **33** | **male** | **1** | **Early-stage** | **ccRCC** |
| **30** | **46** | **male** | **4** | **Advanced-stage** | **ccRCC** |
| **31** | **25** | **male** | **3** | **Advanced-stage** | **ccRCC** |
| **32** | **67** | **female** | **3** | **Advanced-stage** | **ccRCC** |
| **33** | **48** | **male** | **4** | **Advanced-stage** | **ccRCC** |
| **34** | **46** | **male** | **1** | **Early-stage** | **ccRCC** |
| **35** | **72** | **female** | **4** | **Advanced-stage** | **ccRCC** |
| **36** | **75** | **male** | **1** | **Early-stage** | **ccRCC** |
| **37** | **50** | **male** | **1** | **Early-stage** | **ccRCC** |
| **38** | **55** | **male** | **4** | **Advanced-stage** | **ccRCC** |
| **39** | **40** | **male** | **1** | **Early-stage** | **ccRCC** |
| **40** | **64** | **female** | **1** | **Early-stage** | **ccRCC** |
| **41** | **58** | **male** | **1** | **Early-stage** | **ccRCC** |
| **42** | **57** | **male** | **1** | **Early-stage** | **ccRCC** |
| **43** | **52** | **female** | **2** | **Early-stage** | **ccRCC** |
| **44** | **64** | **male** | **1** | **Early-stage** | **ccRCC** |
| **45** | **50** | **male** | **1** | **Early-stage** | **ccRCC** |
| **46** | **48** | **male** | **1** | **Early-stage** | **ccRCC** |
| **47** | **58** | **female** | **1** | **Early-stage** | **ccRCC** |
| **48** | **69** | **male** | **1** | **Early-stage** | **ccRCC** |
| **49** | **75** | **male** | **1** | **Early-stage** | **ccRCC** |
| **50** | **37** | **male** | **1** | **Early-stage** | **ccRCC** |
| **51** | **81** | **male** | **1** | **Early-stage** | **ccRCC** |
| **52** | **60** | **male** | **1** | **Early-stage** | **ccRCC** |
| **53** | **58** | **female** | **1** | **Early-stage** | **ccRCC** |
| **54** | **69** | **female** | **1** | **Early-stage** | **ccRCC** |
| **55** | **35** | **male** | **3** | **Advanced-stage** | **ccRCC** |
| **56** | **65** | **female** | **3** | **Advanced-stage** | **ccRCC** |
| **57** | **72** | **female** | **3** | **Advanced-stage** | **ccRCC** |
| **58** | **71** | **female** | **4** | **Advanced-stage** | **ccRCC** |
| **59** | **58** | **female** | **4** | **Advanced-stage** | **ccRCC** |
| **60** | **54** | **male** | **3** | **Advanced-stage** | **ccRCC** |
| **61** | **59** | **male** | **1** | **Early-stage** | **ccRCC** |
| **62** | **65** | **female** | **3** | **Advanced-stage** | **ccRCC** |
| **63** | **44** | **male** | **4** | **Advanced-stage** | **ccRCC** |
| **64** | **50** | **male** | **4** | **Advanced-stage** | **ccRCC** |
| **65** | **51** | **female** | **4** | **Advanced-stage** | **ccRCC** |
| **66** | **66** | **male** | **3** | **Advanced-stage** | **ccRCC** |
| **67** | **63** | **male** | **3** | **Advanced-stage** | **ccRCC** |
| **68** | **49** | **male** | **3** | **Advanced-stage** | **ccRCC** |
| **69** | **64** | **male** | **4** | **Advanced-stage** | **ccRCC** |
| **70** | **31** | **female** | **3** | **Advanced-stage** | **ccRCC** |
| **71** | **72** | **female** | **1** | **Early-stage** | **ccRCC** |
| **72** | **59** | **female** | **3** | **Advanced-stage** | **ccRCC** |
| **73** | **66** | **female** | **3** | **Advanced-stage** | **ccRCC** |
| **74** | **80** | **male** | **1** | **Early-stage** | **ccRCC** |

Notes: ccRCC, Clear Cell Renal Cell Carcinoma;

The stage_1 column represents the American Joint Committee on Cancer (AJCC) TNM stage;

Early-stage: Stage I or Stage II, Advanced-stage: Stage III or Stage IV.
